# Management of patients with brain metastases from non-small cell lung cancer and adverse prognostic features: multi-national radiation treatment recommendations are heterogeneous

**DOI:** 10.1186/s13014-019-1237-9

**Published:** 2019-02-15

**Authors:** Carsten Nieder, Matthias Guckenberger, Laurie E. Gaspar, Chad G. Rusthoven, Dirk De Ruysscher, Arjun Sahgal, Timothy Nguyen, Anca L. Grosu, Minesh P. Mehta

**Affiliations:** 1 Department of Oncology and Palliative Medicine, Nordland Hospital, 8092 Bodø, Norway; 2 Department of Clinical Medicine, Faculty of Health Sciences, UiT – The Arctic University of Norway, Tromsø, Norway; 3 Department of Radiation Oncology, University Hospital Zurich, University of Zurich, Zurich, Switzerland; 4 Department of Radiation Oncology, University of Colorado School of Medicine, Aurora, CO USA; 5 Department of Radiation Oncology (MAASTRO Clinic), Maastricht University Medical Center, GROW, Maastricht, The Netherlands; 6 Department of Radiation Oncology, Sunnybrook Health Sciences Centre and University of Toronto, Toronto, Canada; 7 Department of Radiation Oncology, University Hospital Freiburg, Freiburg, Germany; 8 Department of Radiation Oncology, Miami Cancer Institute, Miami, FL USA

**Keywords:** Brain metastases, Non-small cell lung cancer, Prognostic factors, Radiosurgery, Whole brain radiation therapy

## Abstract

**Background:**

Different management options exist for patients with brain metastases from non-small cell lung cancer (NSCLC), patients whose treatment with whole brain radiotherapy (WBRT) has become more controversial over the last decade. It is not trivial to find the optimal balance of over- versus undertreatment in these patients. Several recent trials, including the randomized QUARTZ trial now influence the decision to recommend or withhold WBRT for patients with unfavorable prognosis, and similarly, for favorable prognosis patients, the balance between radiosurgery alone or WBRT has become a nuanced decision. Additionally, the availability of intracranially active targeted agent for some subsets of these patients has added another layer of complexity to the decision-making.

**Methods:**

A multinational consortium of expert radiation oncologists was established with the aim of compiling treatment recommendations for challenging scenarios, in this case the choice between optimal supportive care (SC), WBRT and other types of radiation therapy (RT). We distributed 17 cases to 7 radiation oncologists who were allowed to involve coworkers to provide their treatment recommendations. The cases differed in extra- and intracranial disease extent, histology, age and other prognostic factors. Expert recommendations were tabulated with the aim of providing guidance.

**Results:**

Regarding willingness to include the 17 patients in the QUARTZ trial, the rates of trial inclusion were low (range 0/7 to 3/7). Experts not recommending trial inclusion provided their treatment recommendations. These suggestions differed widely for most of the patients. It was not uncommon to see 3 or 4 different recommendations. In general, few (0–2) recommended SC. Some kind of local treatment was suggested by the majority of experts for all 17 patients. Commonly, stereotactic single-fraction radiosurgery (SRS) or stereotactic fractionated radiotherapy (SFRT) were recommended by many experts, also for patients with 5–7 lesions. The highest proportion of recommendations towards WBRT in any patient was 3/7. It was also quite common for patients with multiple metastases of varying size that experts suggested combinations of resection, post-operative SRS/SFRT and SRS/SFRT to intact lesions. Despite recommending active treatment, experts were often willing to include the patients in a hypothetical protocol investigating radiotherapy utilization in the last 30 days of life (assessment of factors predicting early death).

**Conclusions:**

WBRT was infrequently recommended. Even in patients with adverse prognostic features that raised the experts’ awareness of an increased risk for futile treatment near the end of life, SRS/SFRT were more often recommended than optimal supportive care, unless a patient decided to forego active treatment.

**Electronic supplementary material:**

The online version of this article (10.1186/s13014-019-1237-9) contains supplementary material, which is available to authorized users.

## Introduction

The management options for patients with brain metastases from non-small cell lung cancer (NSCLC) have never been more diverse than in the present era [1–3]. While it has been recognized for decades that patients with a solitary lesion might live disease-free for many years after effective treatment, this scenario is uncommon [4]. Most patients present with more than one brain metastasis and have additional extracranial metastases in the liver, adrenal glands and/or bones. If accompanied by reduced performance status and other adverse prognostic features, patients with multiple metastases to multiple organs have been shown repeatedly to have limited survival, e.g., after whole brain radiotherapy (WBRT) [5–7]. However with advanced radiation treatment modalities and evolving systemic treatment options [8–10] survival outcomes of individual patients might be better or worse than anticipated from prognostic models. Parallel to other developments, these models have been refined and now take into account the different histologic and molecular subsets of NSCLC [11–14]. This is of particular relevance, because even patients with poor performance scores and multiple brain metastases and extracranial disease, if harboring an actionable mutation, often respond dramatically to targeted therapies.

Among several current controversies, one relates to the management of patients with adverse prognostic features, where overly aggressive treatment in the final weeks of life is not uncommon [15–17]. In order to shed more light on the role of WBRT in patients not suitable for neurosurgical resection and/or stereotactic radiosurgery (SRS), the prospective randomized QUARTZ trial was performed [18]. Despite initial difficulties in patient accrual [19], 538 patients were recruited from 69 UK and three Australian centers between 2007 and 2014. The protocol required uncertainty in the clinicians’ or patients’ minds about the potential benefit of WBRT and used a non-inferiority design. All patients were offered optimal supportive care (SC) including dexamethasone, which a significant majority received. In the WBRT arm treatment consisted of 5 fractions of 4 Gy each. The primary outcome measure was quality-adjusted life-years (QALYs). Median age was 66 years (range 38–85). The authors concluded that there was no significant difference in QALYs between the two treatment arms (mean 46.4 QALY days for the WBRT arm vs 41.7 QALY days for WBRT and SC, respectively). It is important to remember, however, that the tool used to measure QALY had several questions which pertained to common side effects of steroids, and since these were liberally used in both arms, no QALY difference could realistically have been expected. There was also no significant difference observed in overall survival (hazard ratio 1.06, 95% CI 0.90–1.26), quality of life, or dexamethasone usage between the two groups.

The potential impact of these QUARTZ findings and ongoing controversy around their influence on current management, stimulated our multinational group to gather, compare and contrast our management of brain metastases in patients with NSCLC. This group has previously applied this strategy to other areas of controversy [20, 21].

## Methods

Representatives (radiation oncologists, some with focus on neuro-oncology, others with focus on lung cancer, many with experience in both fields) from seven academic institutions (hereafter referred to as “participants”) were provided with uniform clinical, diagnostic, and therapeutic information, including critical slices of imaging studies. Within their own institution, the participants had permission to present the cases to their collaborators/tumor boards. All institutions had the possibility to treat with WBRT, stereotactic single-fraction radiosurgery (SRS) or stereotactic fractionated radiotherapy (SFRT). Treatment recommendations were then compiled using a standardized template requesting answers to three different questions. Results from each participant were collated and presented back to the group of participants without disclosing the recommendations by any identifying information.

### Clinical information distributed to the participating experts

Clinical baseline data and the critical parts of imaging studies from 17 patients were sent to the seven participants (Table 1). Imaging was provided as per the Additional file 1: Figure S1. All patients had symptomatic brain metastases rather than imaging or screen-detected lesions. Number and size of brain metastases were based on contrast-enhanced brain MRI scans. If these scans were not stored, the corresponding CT scans were sent to the participating experts. The Karnofsky performance status (KPS) was scored at the time of radiation oncology consultation, following the initiation of steroids. Information about PD-L1 status was not available. In cases where the primary tumor was controlled, thoracic CT scans were not sent out. A brief overview of the QUARTZ inclusion/exclusion criteria was also attached to the distributed material. For all patients, prognostic information according to the disease-specific graded prognostic assessment (DS-GPA), molecular DS-GPA, the Barnholtz-Sloan and Zindler nomograms was also provided [12, 14, 15].

**Table 1:** Patient characteristics and (in the last column) answers to the question about inclusion in the randomized QUARTZ study (number of participants who would have felt comfortable enrolling the patient)

Patient nr.	Age in years	NSCLC type	Primary tumor controlled	Other metastases	KPS	Largest lesion size [cm]	Lesion number (MRI)	Time int. [mo]^a^	Mol DS-GPA	DS-GPA	OS pred^b^	QUARTZ incl.
1	69	squamous cell	yes	hep	50	3.8	3	10	1.0	0.5	2.3	2
2	60	poorly diff.	no	lym, adr, hep, ski	50	1.3	2	2	1.0	1.0	2.7	2
3	58	adeno NSCLC	no	hep, oss, oth	60	4.5	3	0	1.0	1.0	3.1	2
4	69	poorly diff.	no	hep, oss	60	2.1	1	8	1.0	1.0	2.6	2
5	61	poorly diff.	no	hep, adr, pul	60	3.3	7	10	0.5	0.0	2.6	2
6	64	squamous cell	no	pul, kidney	70	1.5	1	20	1.0	1.5	2,6	0
7	85	squamous cell	no	pul	60	1.8	1	18	0.5	1.0	< 2.0	2
8	66	adeno NSCLC	no	oss, adr, lym	60	1.0	7	0	0.5	0.0	2.8	1
9	65	poorly diff.	no	pul, oth	50	2.0	2	5	1.0	0.5	2.4	2
10	77	adeno NSCLC	yes	none	50	2.3	6	12	1.0	1.0	3.8	1
11	62	adeno NSCLC	yes	adr	70	2.1	5	29	0.5	0.5	4.1	1
12	78	adeno EGFR mut.	yes	oss	60	4.0	4	54	1.5	0.0	2.6	0
13	64	adeno NSCLC	yes	oss, adr, oth	50	1.4	7	3	0.5	0.0	3.4	2
14	68	adeno NSCLC	no	pul, pleura	60	2.2	3	8	1.0	0.5	2.6	1
15	66	squamous cell	yes	hep, adr, oss	70	1.5	18	8	0.5	0.5	3.0	3
16	65	adeno NSCLC	yes	pul	70	3.3	4	22	1.0	0.5	4.2	0
17	53	adeno NSCLC	no	hep, adr, oss	70	3.4	7	2	0.5	1.0	3.8	1

*NSCLC* non-small cell lung cancer, *EGFR* epidermal growth factor receptor, *hep* liver, *lym* extrathoracic lymph nodes, *adr* adrenal gland, *ski* skin, *oss* bones, *oth* other organs, *pul* lung, *KPS* Karnofsky performance status, *MRI* magnetic resonance imaging scans, *Mol DS-GPA* molecular disease-specific graded prognostic assessment, *OS pred* nomogram-predicted median survival in months

^a^interval between lung cancer diagnosis and presentation with brain metastasis

^b^predicted survival per Barnholtz-Sloan nomogram [14]

All 17 patients had unfavorable prognostic features, reflected in the low DS-GPA and molecular DS-GPA scores (max. 1.5 points, median 0.5 and 1.0, respectively). Five patients had a KPS of 70, the others 50–60. Sixteen patients had extracranial metastases and only one a targetable molecular alteration (EGFR mutation). According to the Barnholtz-Sloan nomogram [14], the median survival that could be expected for this sample was 2.6 months.

Since these 17 cases were real cases, the actual management employed and the survival outcome was known and released to the participants after they provided their input. The actual management employed SC in 10 patients and WBRT (5 fractions of 4 Gy) in 7, respectively. The WBRT group survived for a median of 1.3 months from the first day of radiotherapy. The SC group survived for a median of 1.2 months from imaging diagnosis of brain metastases.

## Results

The first question to the participants from 7 institutions was whether they would have felt comfortable enrolling such a patient in the QUARTZ trial. As shown in Table 1, the likelihood of the seven participants recommending the QUARTZ trial was low (0–3) for each of the 17 patients. While one participant would not have recommended the QUARTZ trial to any of the 17 patients, each of the others would have included at least one of the 17 patients, while one participant would have recommended the trial to 9 of these patients.

The second question to the participants was regarding their treatment recommendation (if the QUARTZ trial would not have been recommended). As shown in Table 2, these recommendations differed widely for most of the patients. It was not uncommon to end up with 3 or 4 different suggestions. In general, few participants (0–2) recommended SC. However, participants acknowledged that patient preferences would be taken into account. Some kind of local treatment approach was recommended by the majority of experts for all 17 patients. Commonly, SRS or SFRT were recommended by many participants. This was true even for patients with more than five brain metastases. In the patient with 18 brain metastases, only two participants recommended WBRT.

**Table 2:** Patient characteristics and treatment recommendations if the QUARTZ trial would not have been recommended

Patient nr.	Age in years	NSCLC type	DS-GPA	Predicted risk of early death^a^	Predicted probability of survival > 12 mo	Actual survival	Treatment recommended	Evidence for advantage from WBRT (level 1b) from the QUARTZ trial based on Forest plot of overall survival
1	69	squamous cell	0.5	97	0	0.4^b^	SFRT: 4 (SC: 1)	No^m^
2	60	poorly diff.	1.0	68	10	0.3^b^	SRS: 4 (SC: 1)	No
3	58	adeno NSCLC	1.0	Not eligible	Not eligible	1.4^b^	SFRT: 3 (SC: 1)^c^	Yes (reason: age < 60)
4	69	poorly diff.	1.0	73	6	1.6^b^	SFRT: 4 (SC: 1)	No
5	61	poorly diff.	0.0	82	3	1.0^b^	SFRT: 4 (SC: 1)^d^	No
6	64	squamous cell	1.5	65	9	2.0^b^	SRS: 5 (SC: 1)	No
7	85	squamous cell	1.0	81	3	0.6^b^	SRS: 6	No
8	66	adeno NSCLC	0.0	41	20	1.8^b^	SRS: 3 (SC: 1)^e^	No
9	65	poorly diff.	0.5	89	3	2.3^b^	SRS: 2 (SC: 1)^f^	No
10	77	adeno NSCLC	1.0	83	4	0.7^b^	SRS: 3 (SC: 1)^g^	No
11	62	adeno NSCLC	0.5	44	18	5.3	SRS: 4 (SC: 1)^h^	No
12	78	adeno EGFR mut.	0.0	95	0	2.6	SFRT: 2 (SC: 2)^i^	No
13	64	adeno NSCLC	0.0	71	8	0.6	SRS: 3 (SC: 1)	No
14	68	adeno NSCLC	0.5	73	6	1.9	SFRT: 3 (SC: 1)^j^	No
15	66	squamous cell	0.5	66	9	1.2	WBRT: 2 (SC: 2)	No
16	65	adeno NSCLC	0.5	84	3	0.5	SFRT: 4 (SC: 1)^k^	No
17	53	adeno NSCLC	1.0	79	4	1.3	Resection: 2 (SC: 2)^l^	Yes (reason: age < 60)

^a^nomogram predicts early death (< 3 months) and survival > 12 months after SRS (Zindler et al. [15])

^b^optimal supportive care (nr. 11–17: whole-brain radiotherapy with 5 fractions of 4 Gy; survival in months)

^c^two would have combined SFRT and SRS, another two had a strong preference for resection of the largest lesion

^d^three would have combined SFRT and SRS

^e^one would have combined SRS and WBRT, two would have given WBRT alone

^f^two had a strong preference for resection of the largest lesion

^g^two would have given WBRT alone

^h^one would have combined SRS and WBRT, one would have combined SFRT and SRS, two would have given WBRT alone

^i^two had a strong preference for resection of the largest lesion, two would have included WBRT as component of care, one would have combined SFRT and SRS

^j^two had a strong preference for resection of the largest lesion, one would have combined SFRT and SRS

^k^one had a strong preference for resection of the cerebellar lesion, one would have combined SFRT and SRS, one would have combined SFRT and WBRT

^l^resection would have followed by SFRT/SRS to other lesions/cavity, one would have combined SFRT and SRS, one would have given WBRT alone

^m^the Forest plot showed improved survival 1) for patients aged younger than 60 years and 2) those with GPA 2.5–3.0

NSCLC: non-small cell lung cancer; EGFR: epidermal growth factor receptor; DS-GPA: disease-specific graded prognostic assessment; WBRT: whole-brain radiotherapy; SFRT: stereotactic fractionated radiotherapy; SRS: stereotactic single-fraction radiosurgery

The highest agreement among six of seven participants was for SRS in patient 7 who had one small brain metastasis, lung metastases and an uncontrolled primary tumor. The second highest agreement among five of seven participants was for SRS in patient 6 who had one small brain metastasis, lung metastases and multiple other extracranial metastases. For patients with multiple brain metastases of varying size, it was also quite common that participants suggested a combination of resection and post-operative SRS/SFRT to the tumor bed and unresected lesions.

The highest number of participants recommending WBRT alone (3 of 7 experts) was for patient 11 with five brain metastases, one in the brainstem, as well as adrenal metastases.

The third and final question asked, “If you had a protocol investigating radiotherapy utilization in the last 30 days of life (assessment of biomarkers, symptoms and quality-of-life items predicting early death), would you recommend it for this patient based on the prognostic information available in the distributed material”? This question related to the often expressed concern that radiation therapy during the last 30 days of life may be futile. As shown in Table 3, the highest agreement, in five of seven participants, was registered in patient 4 who had a large single brainstem metastasis, uncontrolled malignant pleural effusion and other extracranial metastases. In eight other cases, a majority of participants (4 of 7) would have included the patient in this hypothetical protocol. The highest agreement against inclusion (0 of 7) was found in patient 16, who actually had died 0.5 months after start of WBRT. Only 1/7 would have included patient 6, who actually was managed with SC and died 2 months after diagnosis of brain metastases. Patient 11 had the longest survival (5.3 months, treated with WBRT) and would have been included by 2/7 participants. The patient with the second shortest survival after WBRT (0.6 months, patient 13) would have been included by 4/7 participants.

**Table 3:** Patient characteristics and answers to the question about inclusion in a hypothetical study investigating RT utilization in the last 30 days of life (identification of predictive factors, e.g., blood biomarkers and symptom severity)

Patient nr.	Age in years	NSCLC type	Primary tumor controlled	Other metastases	KPS	Largest lesion size [cm]	Lesion number (MRI)	Time int. [mo]^a^	Mol DS-GPA	DS-GPA	OS pred	EoL study incl.
1	69	squamous cell	yes	hep	50	3.8	3	10	1.0	0.5	2.3	4
2	60	poorly diff.	no	lym, adr, hep, ski	50	1.3	2	2	1.0	1.0	2.7	4
3	58	adeno NSCLC	no	hep, oss, oth	60	4.5	3	0	1.0	1.0	3.1	4
4	69	poorly diff.	no	hep, oss	60	2.1	1	8	1.0	1.0	2.6	5
5	61	poorly diff.	no	hep, adr, pul	60	3.3	7	10	0.5	0.0	2.6	4
6	64	squamous cell	no	pul, kidney	70	1.5	1	20	1.0	1.5	2,6	1
7	85	squamous cell	no	pul	60	1.8	1	18	0.5	1.0	< 2.0	2
8	66	adeno NSCLC	no	oss, adr, lym	60	1.0	7	0	0.5	0.0	2.8	4
9	65	poorly diff.	no	pul, oth	50	2.0	2	5	1.0	0.5	2.4	4
10	77	adeno NSCLC	yes	none	50	2.3	6	12	1.0	1.0	3.8	2
11	62	adeno NSCLC	yes	adr	70	2.1	5	29	0.5	0.5	4.1	2
12	78	adeno EGFR mut.	yes	oss	60	4.0	4	54	1.5	0.0	2.6	3
13	64	adeno NSCLC	yes	oss, adr, oth	50	1.4	7	3	0.5	0.0	3.4	4
14	68	adeno NSCLC	no	pul, pleura	60	2.2	3	8	1.0	0.5	2.6	2
15	66	squamous cell	yes	hep, adr, oss	70	1.5	18	8	0.5	0.5	3.0	4
16	65	adeno NSCLC	yes	pul	70	3.3	4	22	1.0	0.5	4.2	0
17	53	adeno NSCLC	no	hep, adr, oss	70	3.4	7	2	0.5	1.0	3.8	3

*NSCLC* non-small cell lung cancer, *EGFR* epidermal growth factor receptor, *hep* liver, *lym* extrathoracic lymph nodes, *adr* adrenal gland, *ski* skin, *oss* bones, *oth* other organs, *pul* lung, *KPS* Karnofsky performance status, *MRI* magnetic resonance imaging scans, *Mol DS-GPA* molecular disease-specific graded prognostic assessment, *OS pred* nomogram-predicted median survival in months, *RT* radiotherapy, *EoL* end of life

^a^interval between lung cancer diagnosis and presentation with brain metastasis

## Discussion

The QUARTZ trial of WBRT plus SC or SC alone had difficulties in patient accrual [19]. The present finding of limited willingness to enroll patients that fulfil the eligibility criteria suggests that experienced clinicians expect little, if any, benefit from palliative WBRT with the 4 Gy × 5 regimen. In fact, the biologically equivalent dose of this regimen is unlikely to provide major tumor shrinkage and long-lasting growth control [22, 23]. Historically, WBRT has long been the most widely used treatment for brain metastases from NSCLC (often 3 Gy × 10), from todays point of view based on sub-optimal evidence from studies that are difficult to translate into current practice (pre-MRI era, limited possibilities to determine the TNM stage correctly, limited possibilities to treat extracranial disease). Opponents of WBRT commonly point out its negative impact on health-related quality-of-life (fatigue, physical functioning, cognitive functioning), even if these effects are often transitory as published by Soffietti et al. who reported a randomized trial of WBRT versus observation after surgical resection or SRS [24]. Since a detailed discussion of detrimental effects from all available publications is beyond the scope of this study, the readers are refered to a recent review [25].

The participants often recommended SRS and SFRT regimens, likely in an attempt to improve symptoms (all 17 patients had symptomatic brain metastases rather than imaging or screening detected lesions) and to prevent imminent death from uncontrolled intracranial disease. A proportion of patients seen for consultation express the wish to prevent neurological symptoms such as paresis, blindness or aphasia, even if they realize that life expectance is short. In addition, in today’s day and age, SRS and SFRT treatment is not particularly time consuming for the patient and frequently well tolerated. In fact SRS has become more convenient to plan and deliver, with minimal imposition of time commitment on the part of the patient, and results in minimal interruption of daily life schedule. However, a prospective head to head comparison of SRS/SFRT and SC, providing firm support for SRS/SFRT in this setting, is not available. In principle, the competing risk of death from thoracic disease and/or extracranial metastases might limit the net benefit of effective local brain-directed treatment. An over-optimistic estimation of patients’ prognosis may partially explain the utilization of radiotherapy near the end of life. Recent studies have tried to quantify utilization rates [16, 26] and have also suggested prognostic models, which may lead to improved decision-making by radiation oncologists [11, 14, 17, 27–29]. However, the perfect prognostic model has yet to be developed.

For the 7 patients managed with WBRT (4 Gy × 5) the median survival was 1.3 months measured from the first day of radiotherapy. This means that several patients received WBRT during the last 30 days of life. Interestingly, many participating experts were aware of this risk and were willing to include at least some of the patients in a hypothetical protocol addressing aspects of radiotherapy utilization in the terminal phase of disease. However, agreement between experts was modest and not necessarily reflective of actual survival. These findings are in agreement with previous research, as reviewed by Hui [30].

Since the design of the QUARTZ trial, eligibility criteria for resection and SRS have evolved. Current guidelines reflect the heterogeneity of the patient population and this results in a need for shared decision-making and discussion of patient preferences if several options are available [31] (Table 4). Also in the present study recommendations differed widely for most of the patients. It was not uncommon to end up with 3 or 4 different suggestions, a finding that underlines how difficult it is to interpret the published studies and to generate evidence-based recommendations. If seeking additional advice from other providers, e.g. second opinion, many patients may receive different treatment proposals, and also actual treatment is likely more stochastic than desirable. In general, few recommendations (0–2) were given towards SC. It was more common for patients with multiple metastases of varying size that experts suggested combinations of resection, post-operative SRS/SFRT and SRS/SFRT to intact lesions. These individualized approaches have the potential to result in long-term survival even in patients with Lung-molGPA 0–1 (the group with poorest survival; four prognostic strata in total), as shown in the dataset published by Sperduto et al. [12]. Median survival was 5.3 and 6.9 months in patients with non-adenocarcinoma and adenocarcinoma, respectively. An important aspect to consider is the mix of patients with low- and high-volume brain metastases. The patients selected for our study were heavily weighted towards unfavorable size and location of the lesions. Furthermore, except for patient 12, they lacked driver mutations for targeted systemic therapy. The presence of such targets (ROS1, ALK, EGFR etc.) complicates decision-making because the optimal role of radiotherapy in the multimodal setting is still under debate. Fortunately, systemic treatment is able to increase survival in eligible patients, sometimes beyond 5 years [35]. There is a clear need for additional prospective studies in this challenging patient population to improve prognostic tools and treatment decisions. An important, recently launched phase III trial (WBRT versus SRS for 4–10 brain metastases) unfortunately has already been closed because of lack of accrual [36], while a different trial is still open (WBRT versus SRS for 5–15 brain metastases; NCT03550391). Probably, inclusion bias is problematic for randomized trials, in particular if one can choose reimbursed treatments without level I evidence.

**Table 4:** Selected guidelines for treatment of brain metastases

Guideline	Ref.	Published	NSCLC specific	Important recommendations and messages
EANO	[31]	2017	No, but contains a NSCLC section with main focus on systemic therapy	The decision regarding whether to employ SRS, SFRT, WBRT, alone or in combination, for patients with multiple brain metastases comes down to clinical discretion, patient preference and logistical considerations with the absolute number of brain metastases becoming less crucial WBRT or best supportive care should be considered for patients with short life expectancy (low KPS score and/or progressive systemic disease)
UK NICE	[32]	2018	No	Consider maximal local therapy with either surgery, SRS or SFRT for people with a single brain metastasis Consider SRS/SFRT for people with multiple brain metastases who have controlled or controllable extracranial disease and KPS of 70 or more; take into account the number and total volume of metastases Do not offer WBRT to people with NSCLC and brain metastases that are not suitable for surgery or SRS/SFRT and have a KPS of under 70
National Norwegian guideline	[33]	2018	Yes	SRS/SFRT should be considered for 1–4 brain metastases If ECOG PS 3–4 SC is recommended, if better PS and > 4 brain metastases WBRT is recommended (4 Gy × 5 or 3 Gy × 10)
Princess Margaret Cancer Centre	[34]	2018	No	The standard of care for patients with brain metastases is currently in a state of flux WBRT is our usual recommendation for patients with > 4–6 brain metastases

*EANO* European Association of Neuro-Oncology, *NSCLC* non-small cell lung cancer, *SRS* stereotactic radiosurgery, *SFRT* stereotactic fractionated radiotherapy, *WBRT* whole-brain radiotherapy, *KPS* Karnofsky performance status, *UK NICE* United Kingdom National Institute for Health and Care Excellence, *ECOG* Eastern Cooperative Oncology Group, *SC* supportive care

## Conclusions

The frequency of recommendations for WBRT was low, despite technologically advanced variants that include hippocampal sparing or simultaneous integrated boost. Even in patients with adverse prognostic features that raised the experts’ awareness of an increased risk of futile treatment near the end of life, SRS or SFRT were more often recommended than SC, unless a patient clearly expressed the desire to forego active treatment.

## Additional file

Additional file 1: **Figure S1.** Representative computed tomography and magnetic resonance imaging scans distributed to the participants. (PDF 3034 kb)

## Abbreviations

CT: Computed tomography
MRI: Magnetic resonance imaging
NSCLC: Non-small cell lung cancer
SC: Optimal supportive care
SFRT: Stereotactic fractionated radiotherapy
SRS: Stereotactic radiosurgery
WBRT: Whole-brain radiotherapy

## References

[1] Hartgerink D, van der Heijden B, De Ruysscher D, Postma A, Ackermans L, Hoeben A, Anten M, Lambin P, Terhaag K, Jochems A, Dekker A, Schoenmaekers J, Hendriks L, Zindler J (2018). Stereotactic radiosurgery in the management of patients with brain metastases of non-small cell lung cancer: indications, decision tools and future directions. Front Oncol.

[2] Frega S, Bonanno L, Guarneri V, Conte P, Pasello G (2018). Therapeutic perspectives for brain metastases in non-oncogene addicted non-small cell lung cancer (NSCLC): towards a less dismal future?. Crit Rev Oncol Hematol.

[3] Trifiletti DM, Sheehan JP, Grover S, Dutta SW, Rusthoven CG, Kavanagh BD, Sahgal A, Showalter TN (2017). National trends in radiotherapy for brain metastases at time of diagnosis of non-small cell lung cancer. J Clin Neurosci.

[4] Nieder C, Hintz M, Oehlke O, Bilger A, Grosu AL (2017). The TNM 8 M1b and M1c classification for non-small cell lung cancer in a cohort of patients with brain metastases. Clin Transl Oncol.

[5] Jeene PM, de Vries KC, van Nes JGH, Kwakman JJM, Wester G, Rozema T, Braam PM, Zindler JD, Koper P, Nuyttens JJ, Vos-Westerman HA, Schmeets I, Niël CGHJ, Hutschemaekers S, van der Linden YM, Verhoeff JJC, Stalpers LJA (2018). Survival after whole brain radiotherapy for brain metastases from lung cancer and breast cancer is poor in 6325 Dutch patients treated between 2000 and 2014. Acta Oncol.

[6] Tsakonas G, Hellman F, Gubanski M, Friesland S, Tendler S, Lewensohn R, Ekman S, de Petris L (2018). Prognostic factors affecting survival after whole brain radiotherapy in patients with brain metastasized lung cancer. Acta Oncol.

[7] Rancoule C, Vallard A, Guy JB, Espenel S, Diao P, Chargari C, Magné N (2017). Brain metastases from non-small cell lung carcinoma: changing concepts for improving patients' outcome. Crit Rev Oncol Hematol..

[8] Oehlke O, Wucherpfennig D, Fels F, Frings L, Egger K, Weyerbrock A, Prokic V, Nieder C, Grosu AL (2015). Whole brain irradiation with hippocampal sparing and dose escalation on multiple brain metastases: local tumour control and survival. Strahlenther Onkol.

[9] Sperduto PW, Wang M, Robins HI, Schell MC, Werner-Wasik M, Komaki R, Souhami L, Buyyounouski MK, Khuntia D, Demas W, Shah SA, Nedzi LA, Perry G, Suh JH, Mehta MP (2013). A phase 3 trial of whole brain radiation therapy and stereotactic radiosurgery alone versus WBRT and SRS with temozolomide or erlotinib for non-small cell lung cancer and 1 to 3 brain metastases: radiation therapy oncology group 0320. Int J Radiat Oncol Biol Phys.

[10] Sperduto PW, Yang TJ, Beal K, Pan H, Brown PD, Bangdiwala A, Shanley R, Yeh N, Gaspar LE, Braunstein S, Sneed P, Boyle J, Kirkpatrick JP, Mak KS, Shih HA, Engelman A, Roberge D, Arvold ND, Alexander B, Awad MM, Contessa J, Chiang V, Hardie J, Ma D, Lou E, Sperduto W, Mehta MP (2016). The effect of gene alterations and tyrosine kinase inhibition on survival and cause of death in patients with adenocarcinoma of the lung and brain metastases. Int J Radiat Oncol Biol Phys.

[11] Nieder C, Mehta MP, Geinitz H, Grosu AL (2018). Prognostic and predictive factors in patients with brain metastases from solid tumors: a review of published nomograms. Crit Rev Oncol Hematol..

[12] Sperduto PW, Yang TJ, Beal K, Pan H, Brown PD, Bangdiwala A, Shanley R, Yeh N, Gaspar LE, Braunstein S, Sneed P, Boyle J, Kirkpatrick JP, Mak KS, Shih HA, Engelman A, Roberge D, Arvold ND, Alexander B, Awad MM, Contessa J, Chiang V, Hardie J, Ma D, Lou E, Sperduto W, Mehta MP (2017). Estimating survival in patients with lung cancer and brain metastases: an update of the graded prognostic assessment for lung cancer using molecular markers (lung-molGPA). JAMA Oncol.

[13] Nieder C, Hintz M, Oehlke O, Bilger A, Grosu AL (2017). Validation of the graded prognostic assessment for lung cancer with brain metastases using molecular markers (lung-molGPA). Radiat Oncol.

[14] Barnholtz-Sloan JS, Yu C, Sloan AE, Vengoechea J, Wang M, Dignam JJ, Vogelbaum MA, Sperduto PW, Mehta MP, Machtay M, Kattan MW (2012). A nomogram for individualized estimation of survival among patients with brain metastasis. Neuro-Oncology.

[15] Zindler JD, Jochems A, Lagerwaard FJ, Beumer R, Troost EGC, Eekers DBP, Compter I, van der Toorn PP, Essers M, Oei B, Hurkmans CW, Bruynzeel AME, Bosmans G, Swinnen A, Leijenaar RTH, Lambin P (2017). Individualized early death and long-term survival prediction after stereotactic radiosurgery for brain metastases of non-small cell lung cancer: two externally validated nomograms. Radiother Oncol.

[16] Ryoo JJ, Batech M, Zheng C, Kim RW, Gould MK, Kagan AR, Lien WW (2017). Radiotherapy for brain metastases near the end of life in an integrated health care system. Ann Palliat Med.

[17] Nieder C, Norum J, Dalhaug A, Aandahl G, Engljähringer K (2013). Best supportive care in patients with brain metastases and adverse prognostic factors: development of improved decision aids. Support Care Cancer.

[18] Mulvenna P, Nankivell M, Barton R, Faivre-Finn C, Wilson P, McColl E, Moore B, Brisbane I, Ardron D, Holt T, Morgan S, Lee C, Waite K, Bayman N, Pugh C, Sydes B, Stephens R, Parmar MK, Langley RE (2016). Dexamethasone and supportive care with or without whole brain radiotherapy in treating patients with non-small cell lung cancer with brain metastases unsuitable for resection or stereotactic radiotherapy (QUARTZ): results from a phase 3, non-inferiority, randomised trial. Lancet.

[19] Langley RE, Stephens RJ, Nankivell M, Pugh C, Moore B, Navani N, Wilson P, Faivre-Finn C, Barton R, Parmar MK, Mulvenna PM, QUARTZ Investigators (2013). Interim data from the Medical Research Council QUARTZ trial: does whole brain radiotherapy affect the survival and quality of life of patients with brain metastases from non-small cell lung cancer?. Clin Oncol (R Coll Radiol)..

[20] Nieder C, Gaspar LE, De Ruysscher D, Guckenberger M, Mehta MP, Rusthoven CG, Sahgal A, Gkika E (2018). Repeat reirradiation of the spinal cord: multi-national expert treatment recommendations. Strahlenther Onkol.

[21] Nieder C, De Ruysscher D, Gaspar LE, Guckenberger M, Mehta MP, Cheung P, Sahgal A (2017). Reirradiation of recurrent node-positive non-small cell lung cancer after previous stereotactic radiotherapy for stage I disease: a multi-institutional treatment recommendation. Strahlenther Onkol.

[22] Nieder C, Berberich W, Nestle U, Niewald M, Walter K, Schnabel K (1995). Relation between local result and total dose of radiotherapy for brain metastases. Int J Radiat Oncol Biol Phys.

[23] Nieder C, Nestle U, Walter K, Niewald M, Schnabel K (1998). Dose/effect relationships for brain metastases. J Cancer Res Clin Oncol.

[24] Soffietti R, Kocher M, Abacioglu UM, Villa S, Fauchon F, Baumert BG, Fariselli L, Tzuk-Shina T, Kortmann RD, Carrie C, Ben Hassel M, Kouri M, Valeinis E, van den Berge D, Mueller RP, Tridello G, Collette L, Bottomley A (2013). A European Organisation for Research and Treatment of Cancer phase III trial of adjuvant whole-brain radiotherapy versus observation in patients with one to three brain metastases from solid tumors after surgical resection or radiosurgery: quality-of-life results. J Clin Oncol.

[25] Brown PD, Ahluwalia MS, Khan OH, Asher AL, Wefel JS, Gondi V (2018). Whole-brain radiotherapy for brain metastases: evolution or revolution?. J Clin Oncol.

[26] Spencer K, Morris E, Dugdale E, Newsham A, Sebag-Montefiore D, Turner R, Hall G, Crellin A (2015). 30 day mortality in adult palliative radiotherapy--a retrospective population based study of 14,972 treatment episodes. Radiother Oncol.

[27] Agarwal JP, Chakraborty S, Laskar SG, Mummudi N, Patil VM, Upasani M, Prabhash K, Noronha V, Joshi A, Purandare N, Tandon S, Arora J, Badhe R (2018). Applying the QUARTZ trial results in clinical practice: development of a prognostic model predicting poor outcomes for non-small cell lung cancers with brain metastases. Clin Oncol (R Coll Radiol).

[28] Jung H, Sinnarajah A, Enns B, Voroney JP, Murray A, Pelletier G, Wu JS (2013). Managing brain metastases patients with and without radiotherapy: initial lessons from a team-based consult service through a multidisciplinary integrated palliative oncology clinic. Support Care Cancer.

[29] Nieder C, Norum J, Hintz M, Grosu AL (2017). Short survival time after palliative whole brain radiotherapy: can we predict potential overtreatment by use of a nomogram?. J Cancer.

[30] Hui D (2015). Prognostication of survival in patients with advanced cancer: predicting the unpredictable?. Cancer Control.

[31] Soffietti R, Abacioglu U, Baumert B, Combs SE, Kinhult S, Kros JM, Marosi C, Metellus P, Radbruch A, Villa Freixa SS, Brada M, Carapella CM, Preusser M, Le Rhun E, Rudà R, Tonn JC, Weber DC, Weller M (2017). Diagnosis and treatment of brain metastases from solid tumors: guidelines from the European Association of Neuro-Oncology (EANO). Neuro-Oncology.

[32] https://www.nice.org.uk/guidance/ng99/chapter/Recommendations#management-of-confirmed-brain-metastases. Accessed 25 Jan 2019.

[33] https://www.helsebiblioteket.no/retningslinjer/lungekreft/palliativ-livsforlengende/palliativ-str%C3%A5lebehandling. Accessed 25 Jan 2019.

[34] https://www.uhn.ca/PrincessMargaret/Health_Professionals/Programs_Departments/Documents/CPG_CNS_BrainMetastases.pdf. Accessed 25 Jan 2019.

[35] Bulbul A, Forde PM, Murtuza A, Woodward B, Yang H, Bastian I, Ferguson PK, Lopez-Diaz F, Ettinger DS, Husain H (2018). Systemic treatment options for brain metastases from non-small-cell lung cancer. Oncology (Williston Park).

[36] Zindler JD, Bruynzeel AME, Eekers DBP, Hurkmans CW, Swinnen A, Lambin P (2017). Whole brain radiotherapy versus stereotactic radiosurgery for 4-10 brain metastases: a phase III randomised multicentre trial. BMC Cancer.

