# Supplementary material for: Management of patients with brain metastases from non-small cell lung cancer and adverse prognostic features: multi-national radiation treatment recommendations are heterogeneous
Source: Radiat Oncol. 2019 Feb 15;14:33. doi: 10.1186/s13014-019-1237-9 (PMC6377775; doi:10.1186/s13014-019-1237-9)

QUARTZ (Mulvenna et al. [17]) randomized between WBRT 4 Gy x5 and BSC

Inclusion and exclusion criteria:

- **Uncertainty in the clinicians' or patients' minds about the potential benefit of WBRT**
- **Unsuitable for either surgery or stereotactic radiotherapy**
- **No previous radiotherapy to the brain**

Patient characteristics from Table 1 of the publication:

- **Age 38-85 years**
- **30% had 1 brain metastasis**
- **KPS was 50-90 with very few exceptions**
- **Patients with or without extracranial metastases were eligible**

Patient 1: 3 brain metastases (same side, close to each other), lung controlled, liver metastases

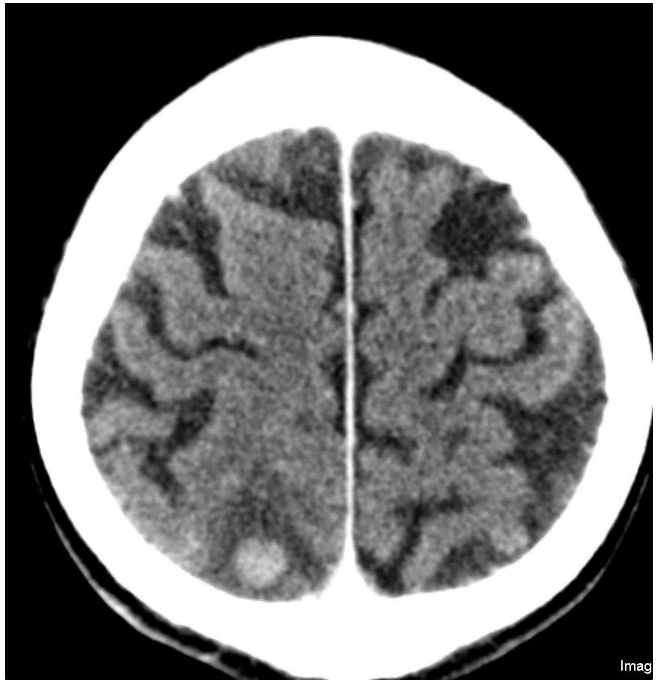

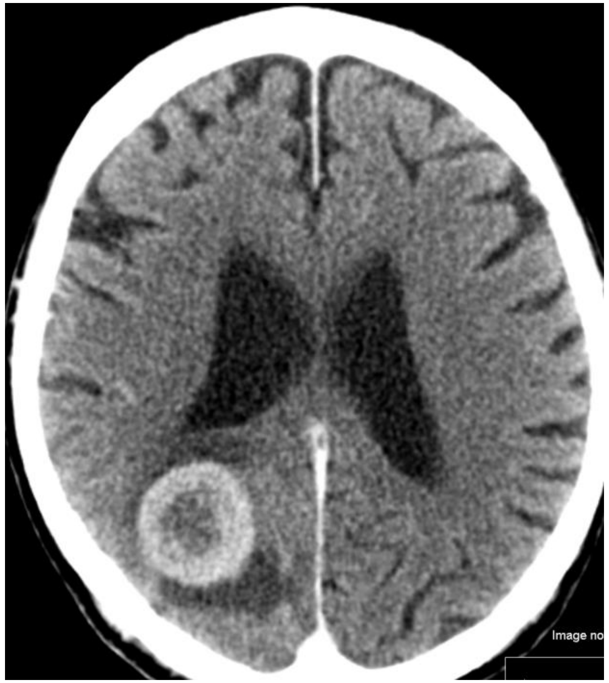

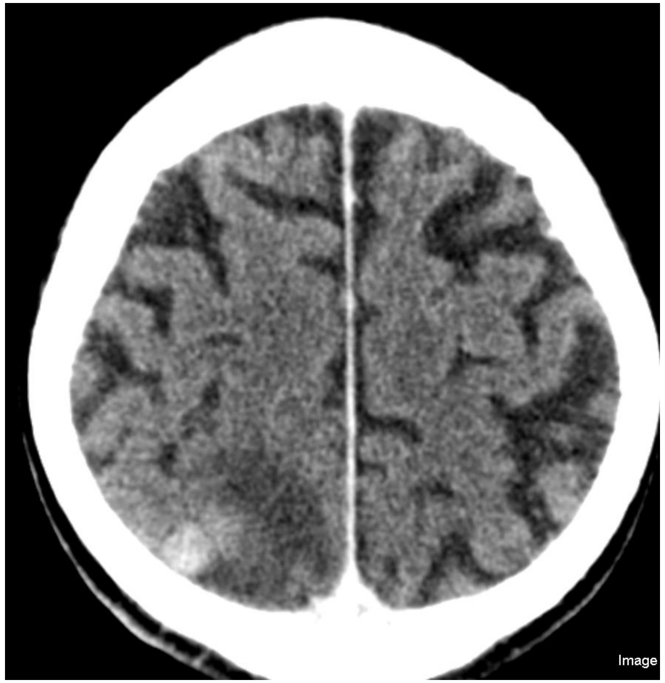

Patient 2: 2 brain metastases, lung uncontrolled local stage IIIA, several other distant metastases

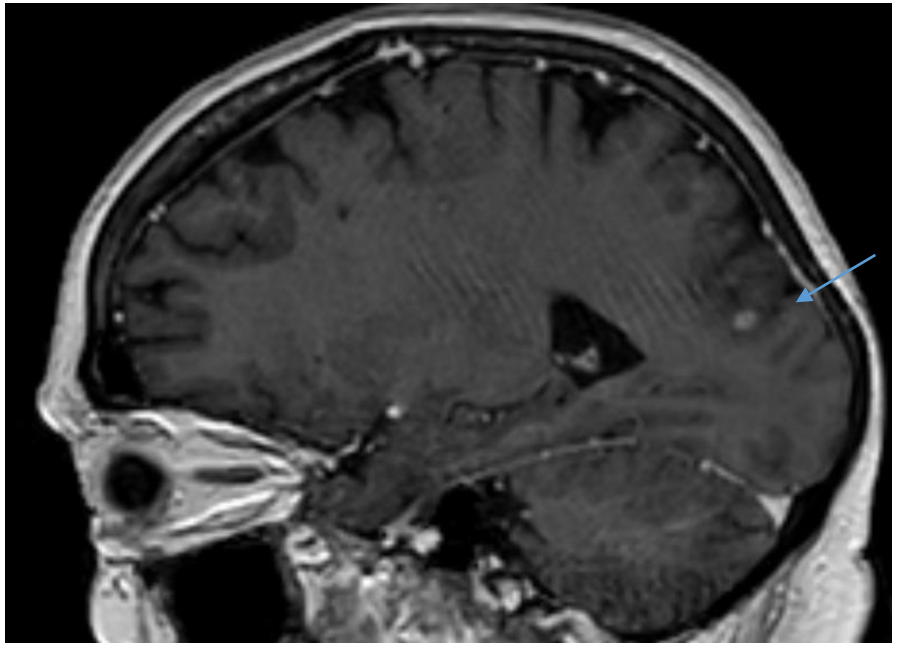

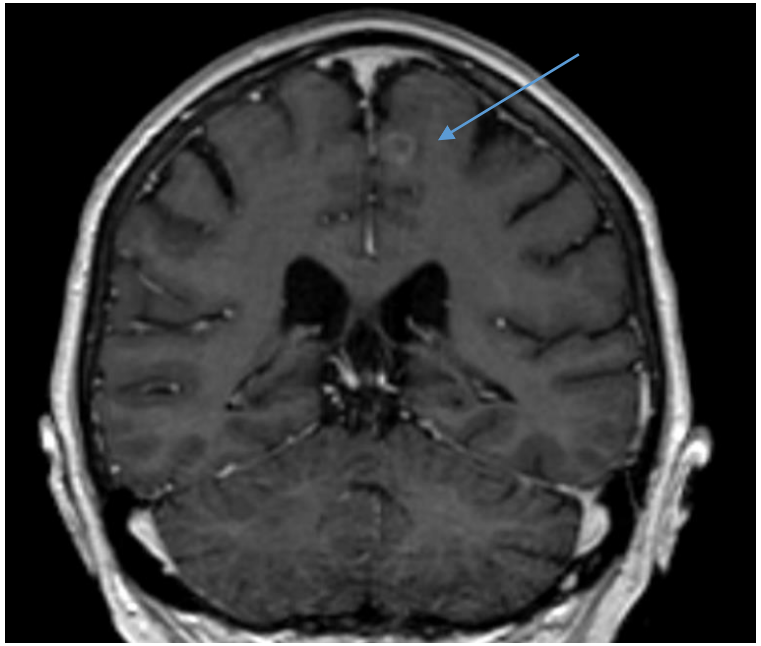

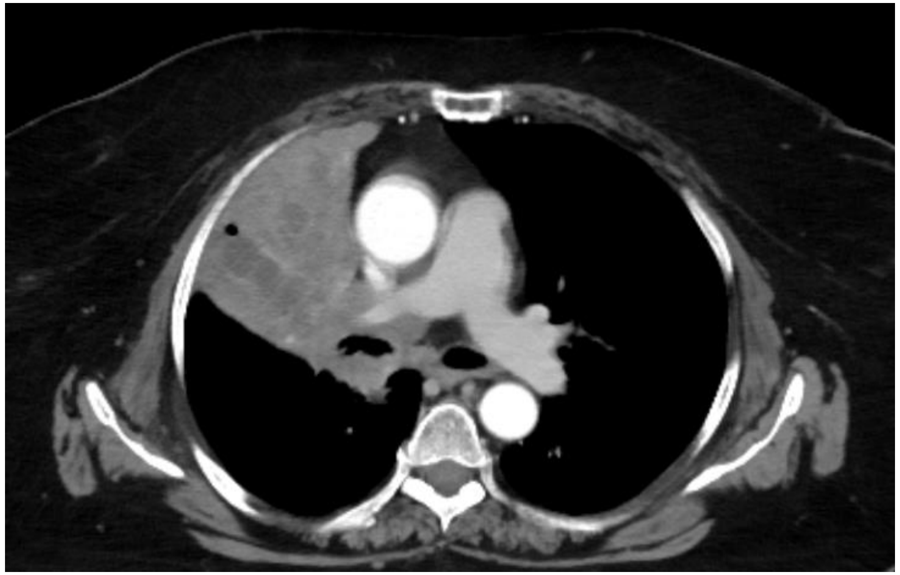

Patient 3: 3 brain metastases, lung uncontrolled local stage IIIA, several other distant metastases

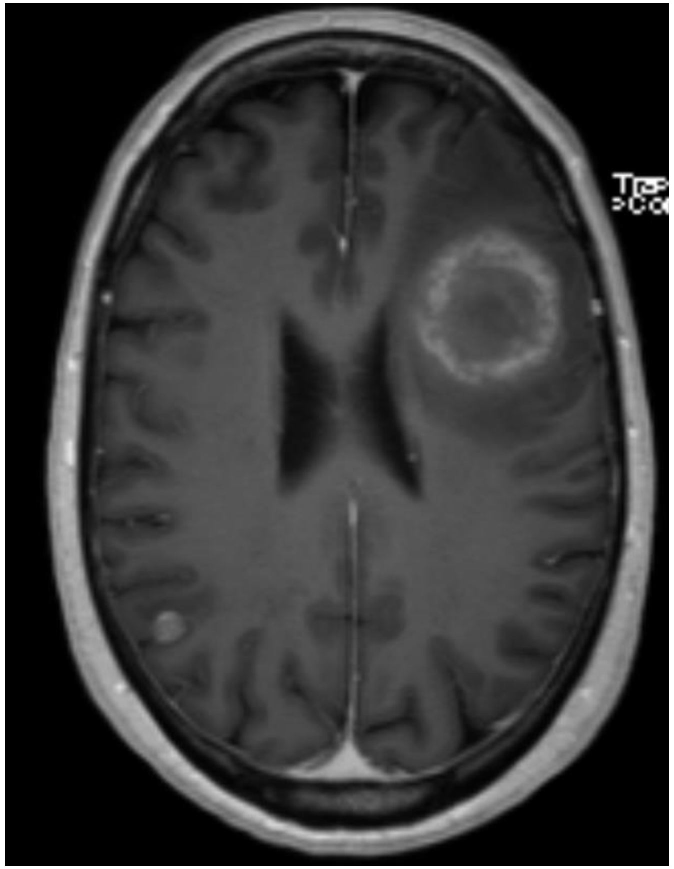

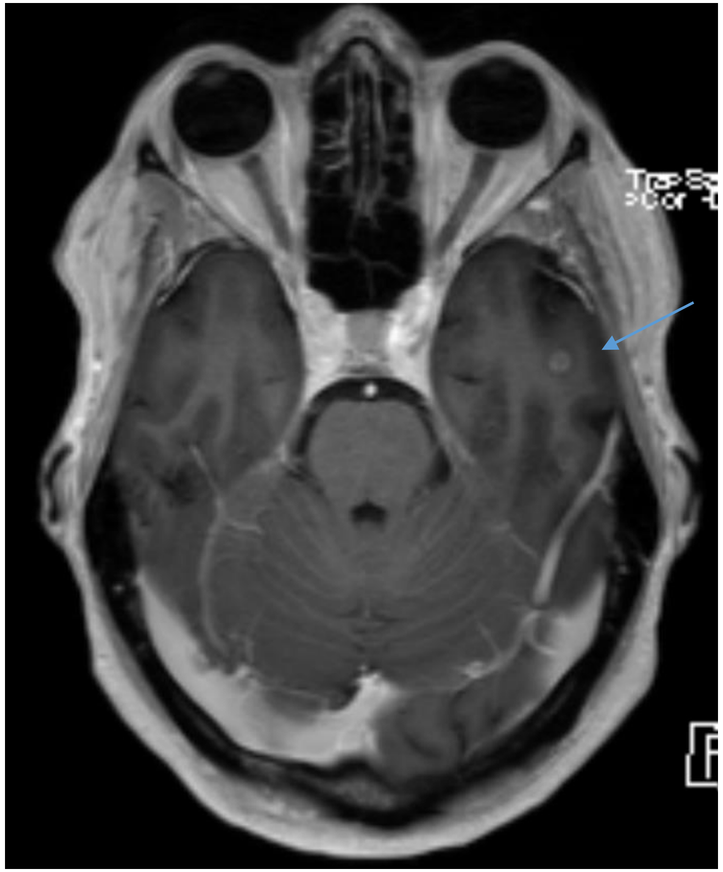

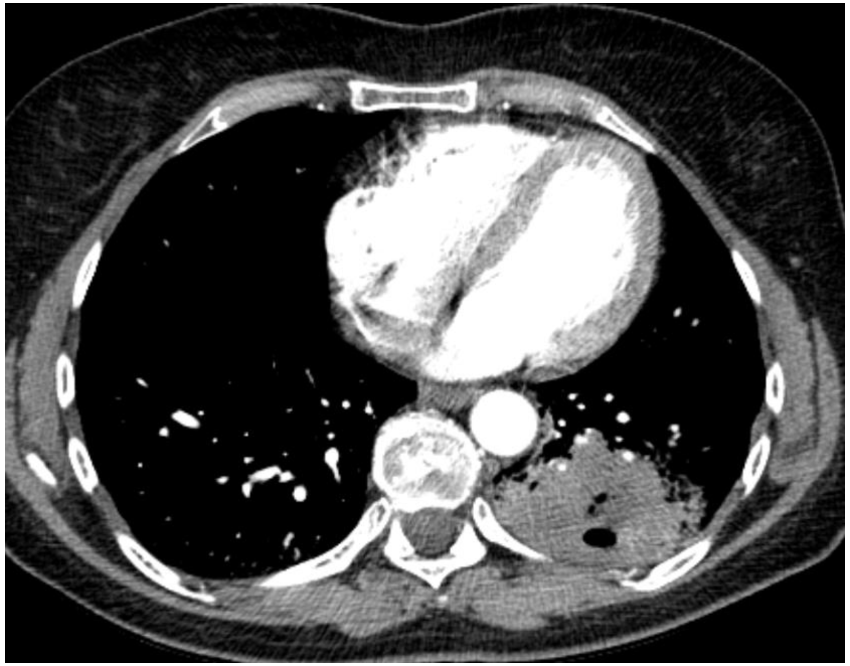

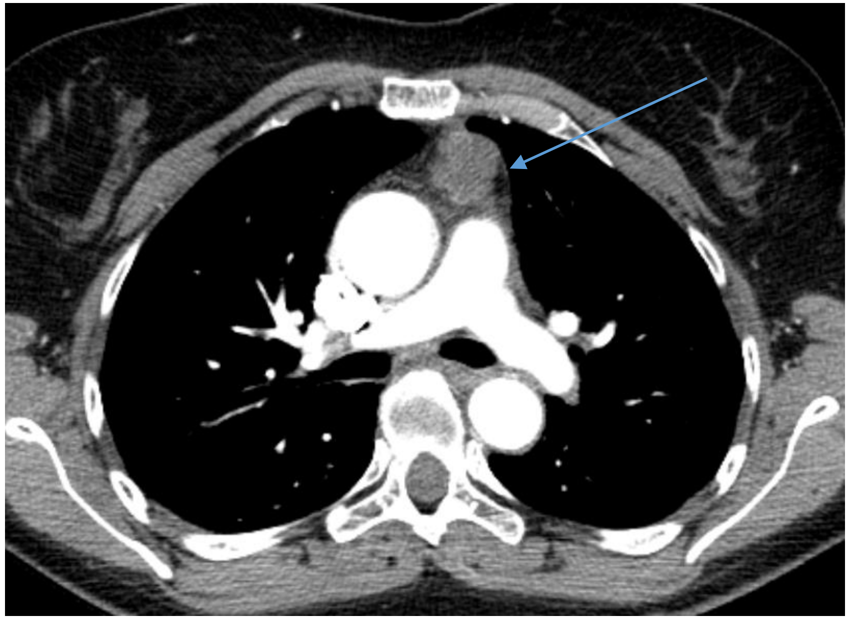

Patient 4: 1 brain metastasis, lung uncontrolled with malignant pleural effusion, several other distant metastases

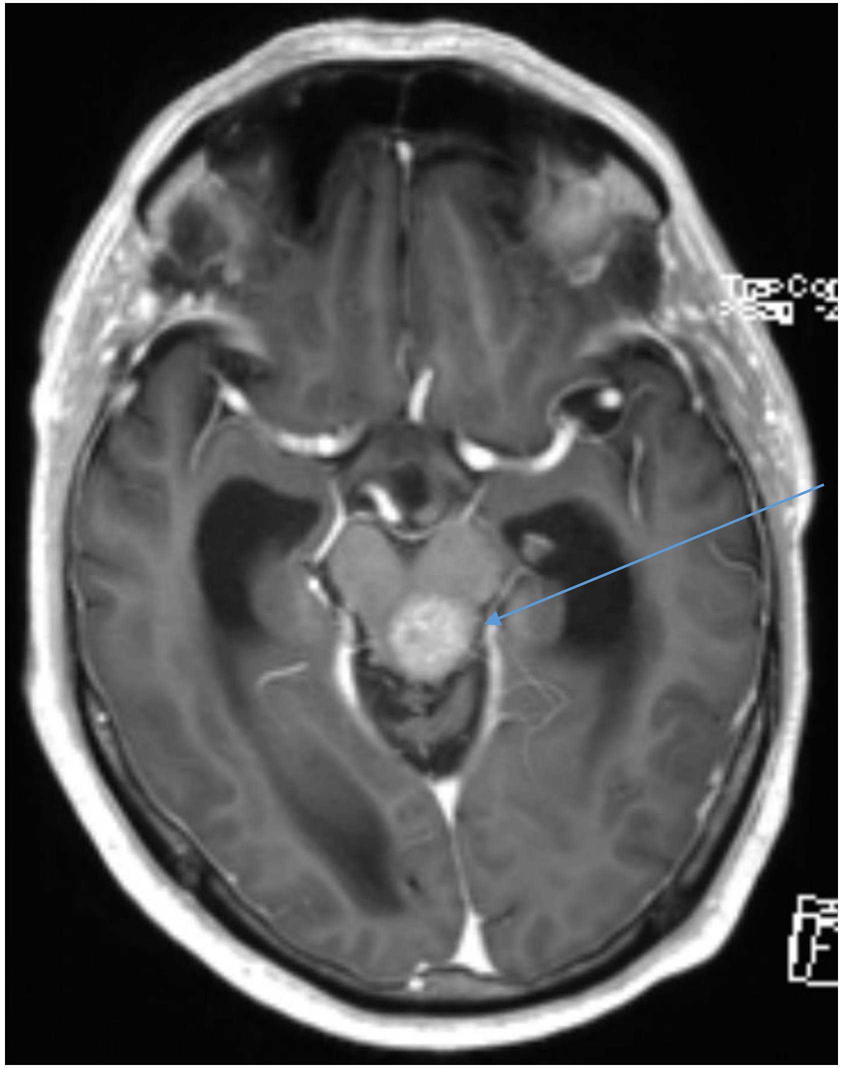

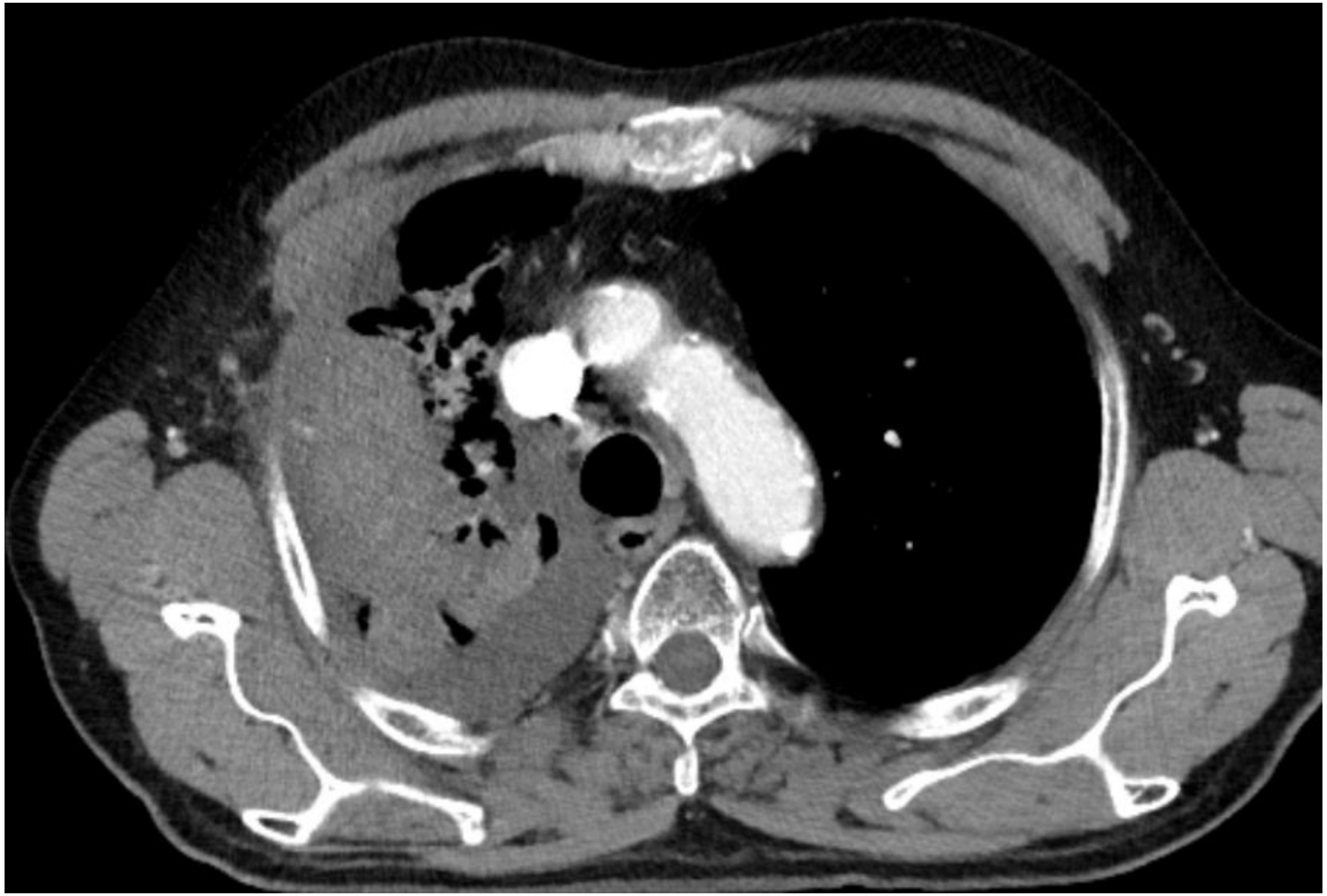

Patient 5: 7 brain metastases (6 supratentorial on both sides), lung uncontrolled local stage IIIA, several other distant metastases

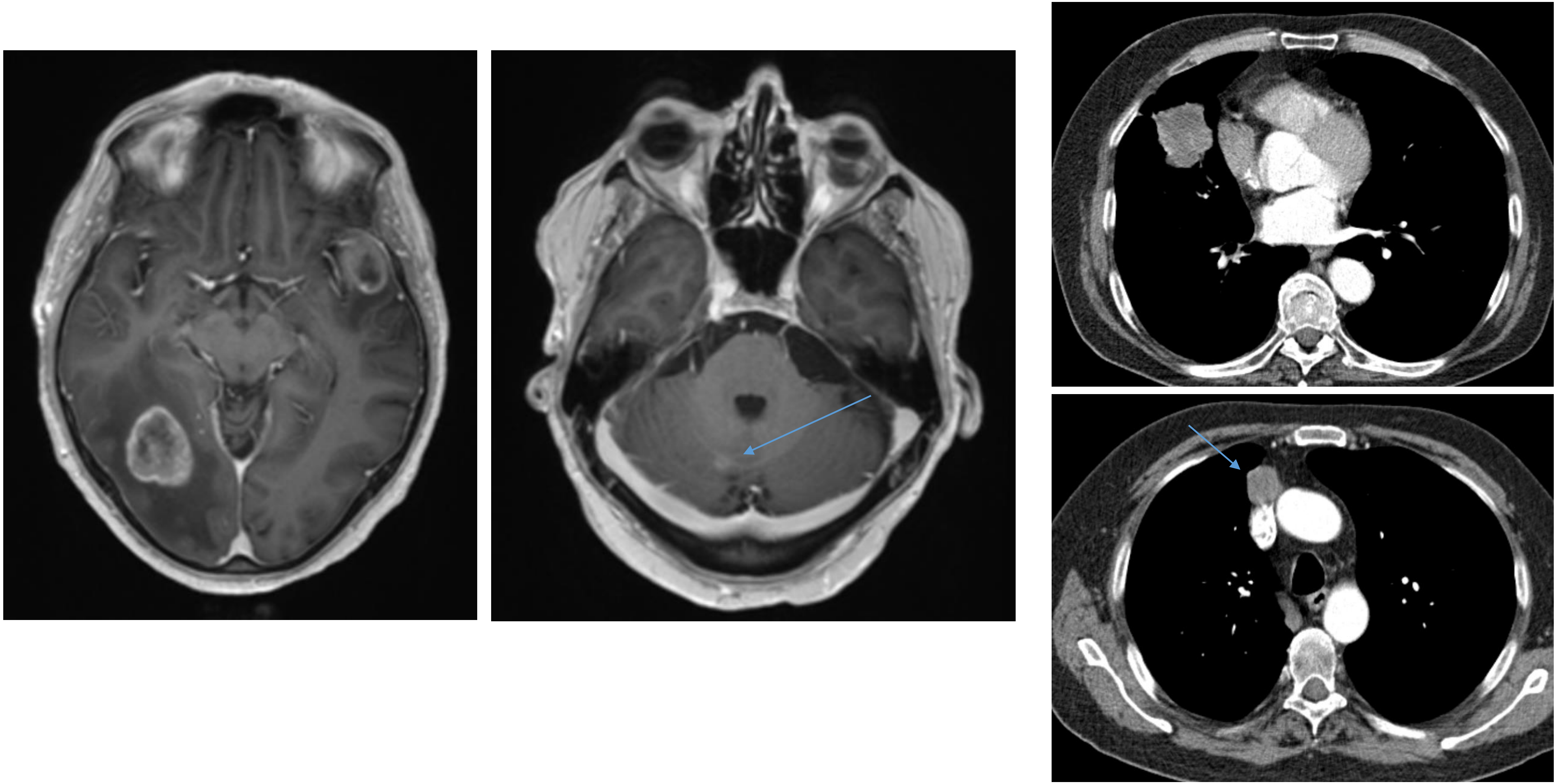

Patient 6: 1 brain metastasis, lung uncontrolled numerous small lesions, several other distant metastases

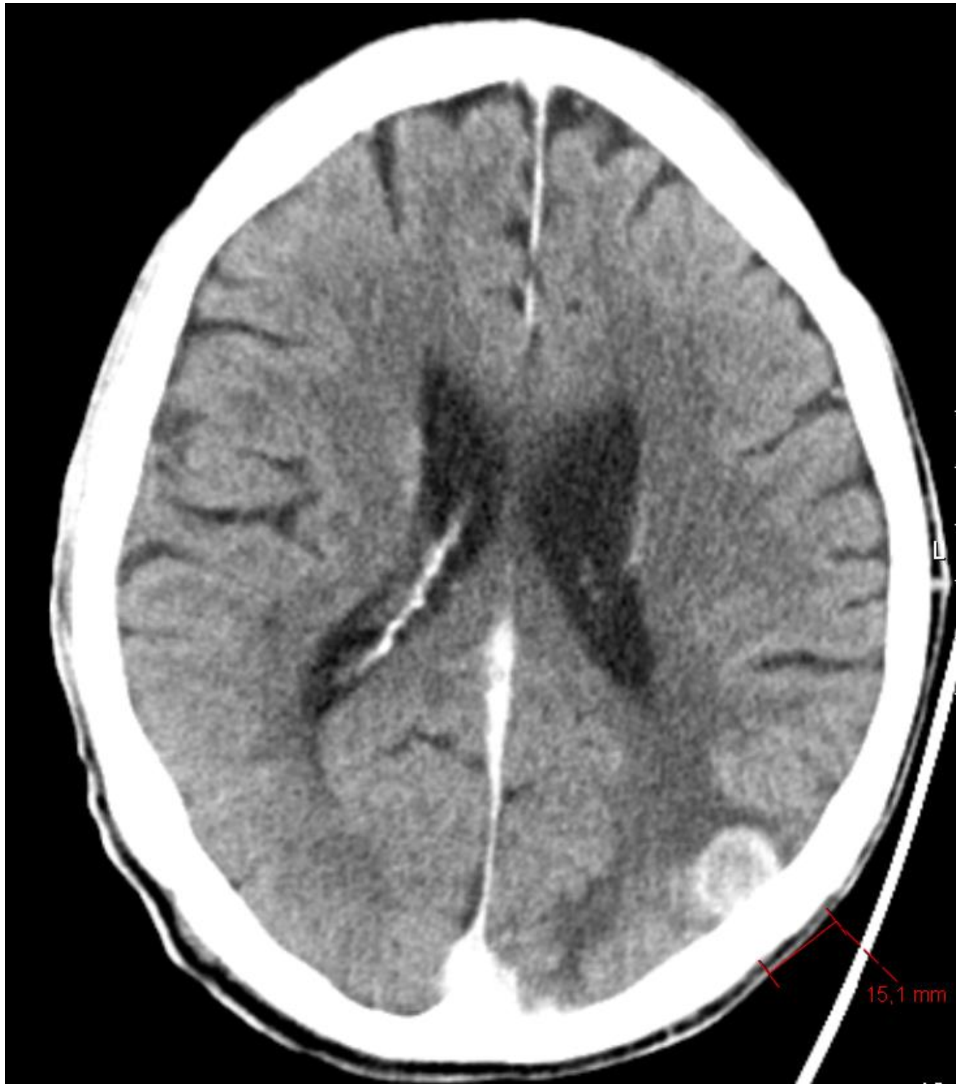

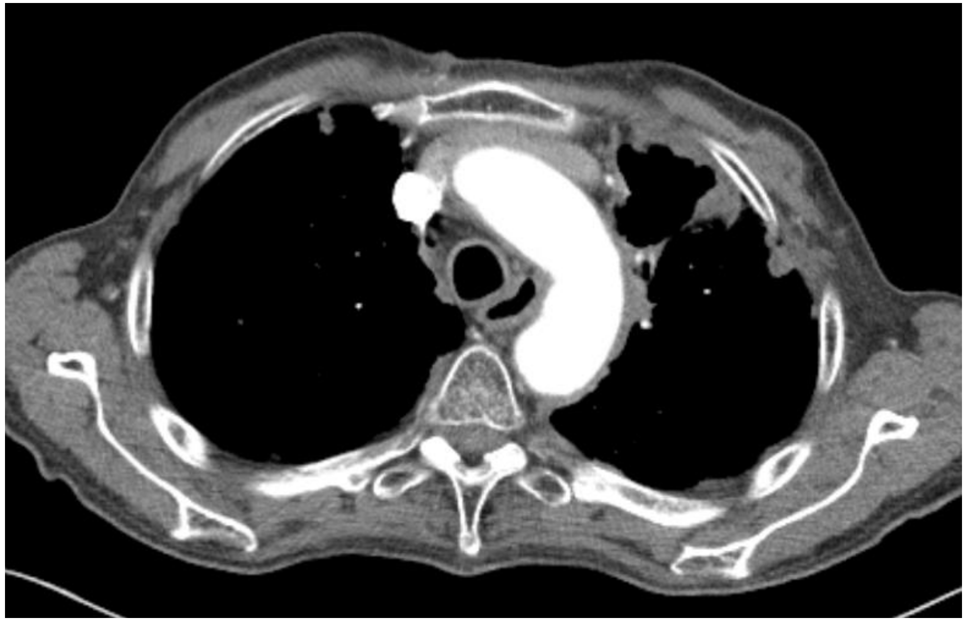

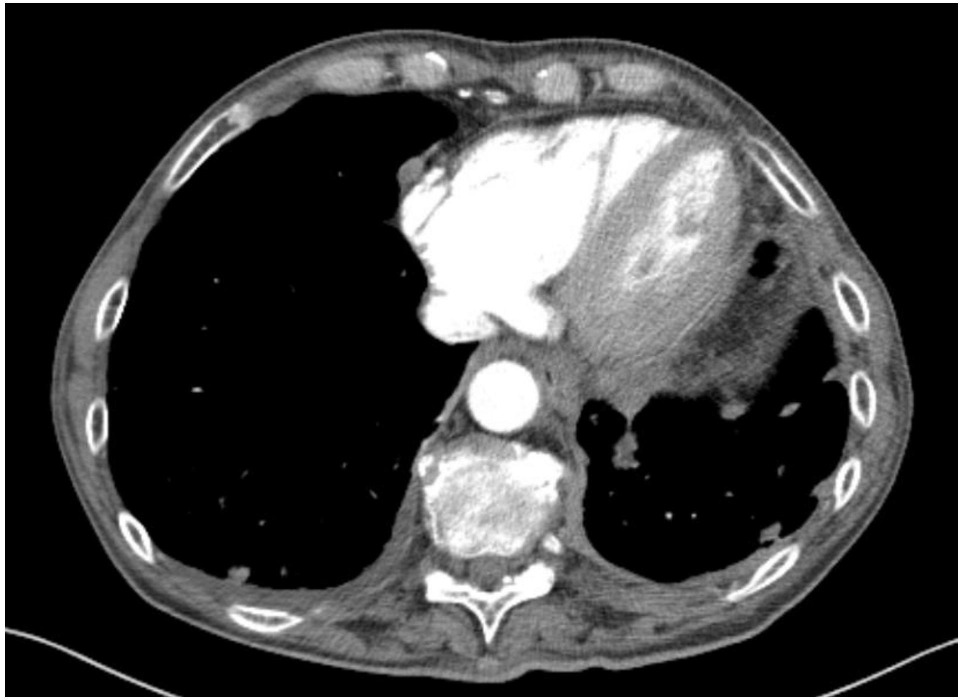

Patient 7: 1 brain metastasis, lung uncontrolled local stage IIIA, additional lung metastases

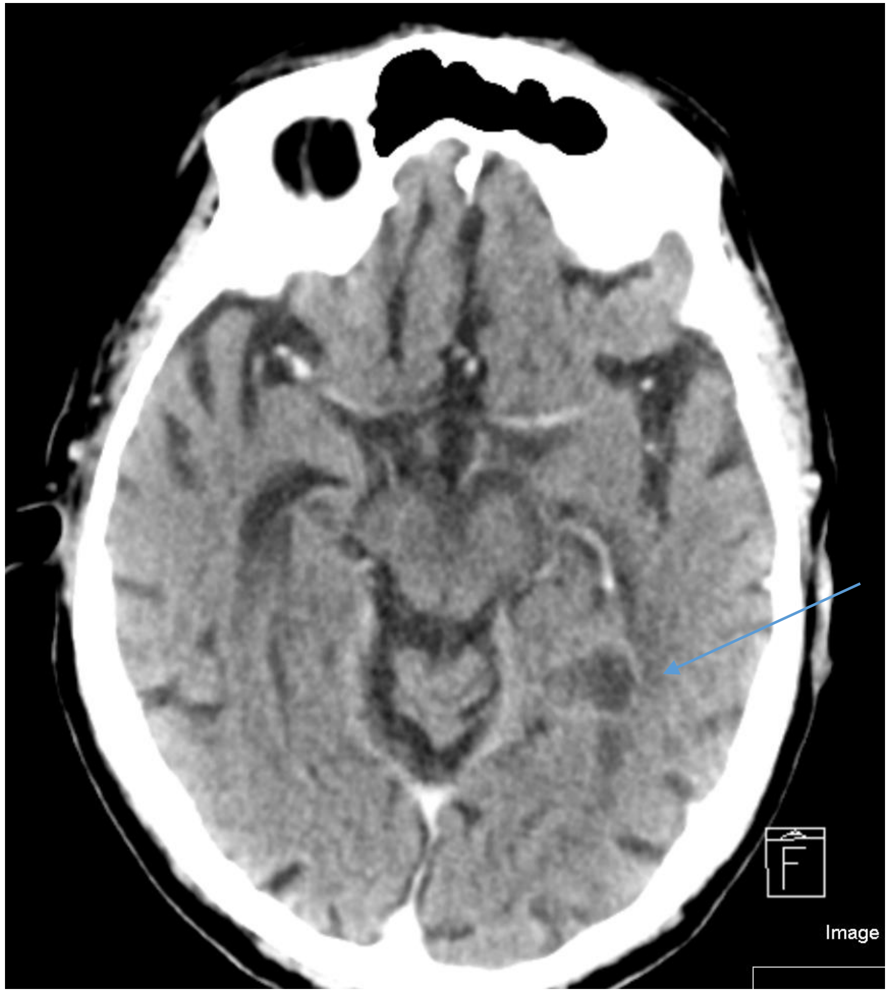

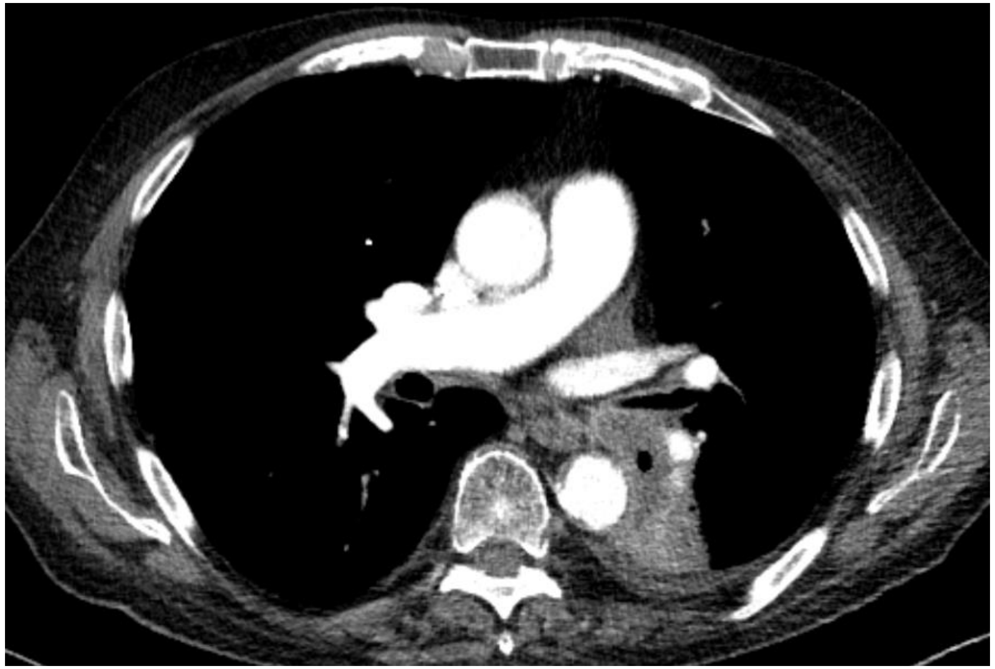

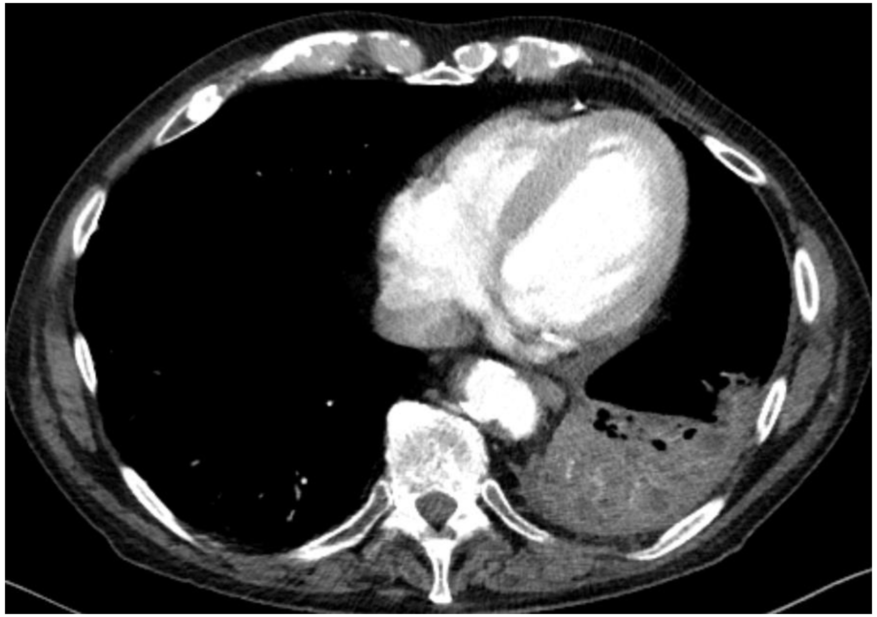

Patient 8: 7 brain metastases (all supratentorial), lung uncontrolled local stage IIIB, several other distant metastases

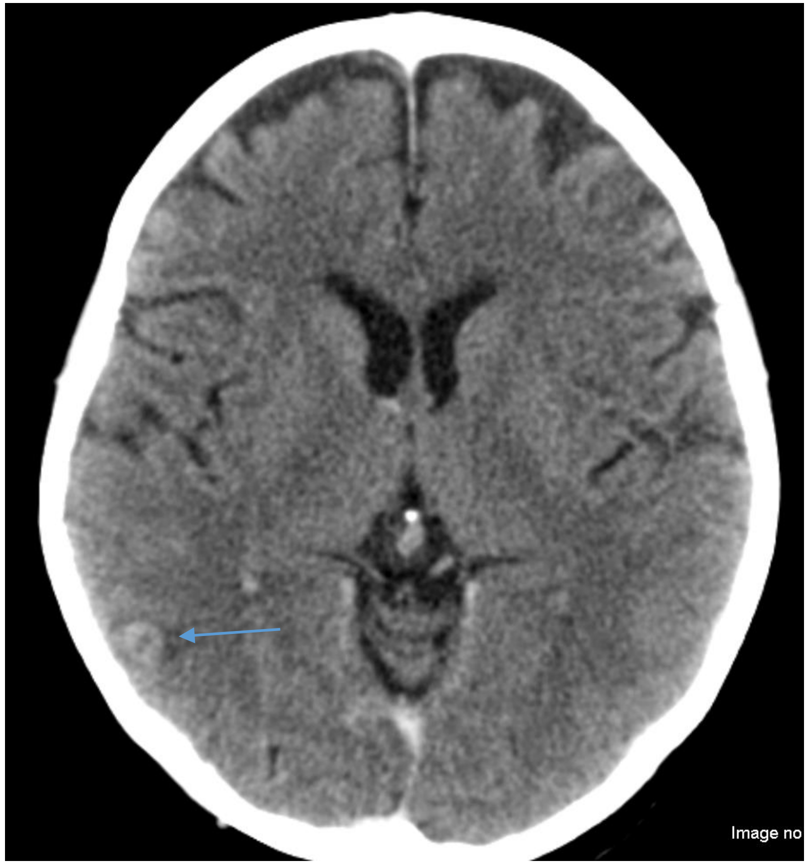

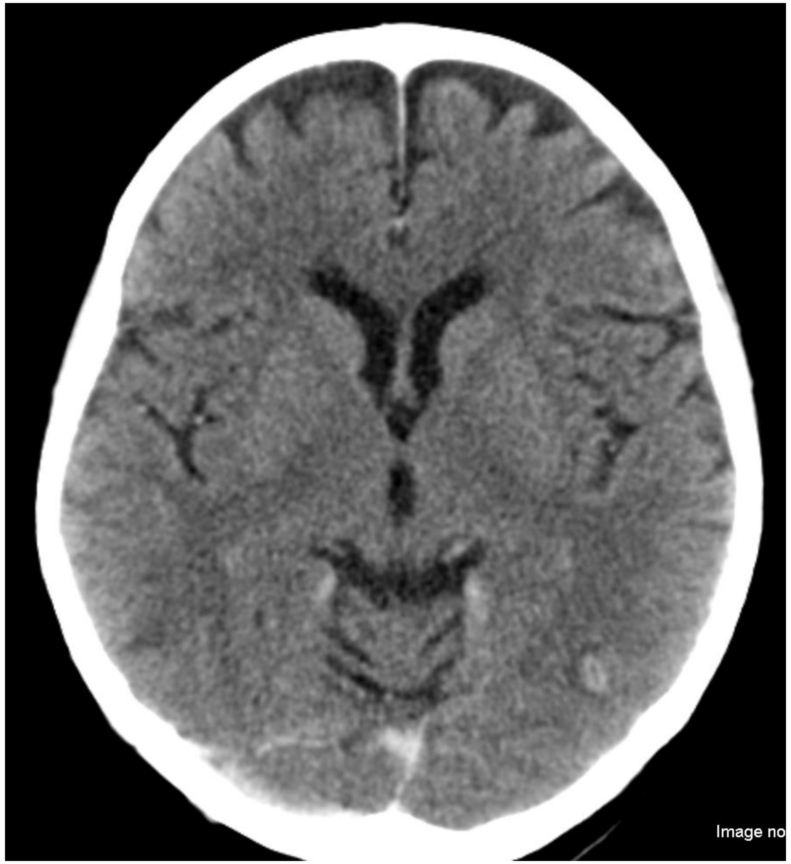

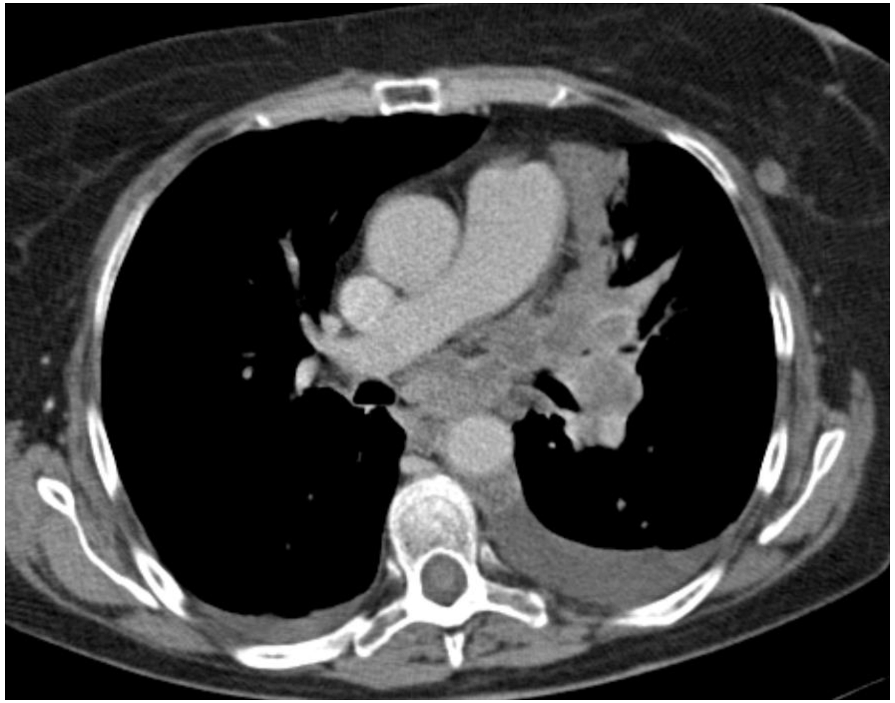

Patient 9: 2 brain metastases, lung uncontrolled local stage IIIB, several other distant metastases

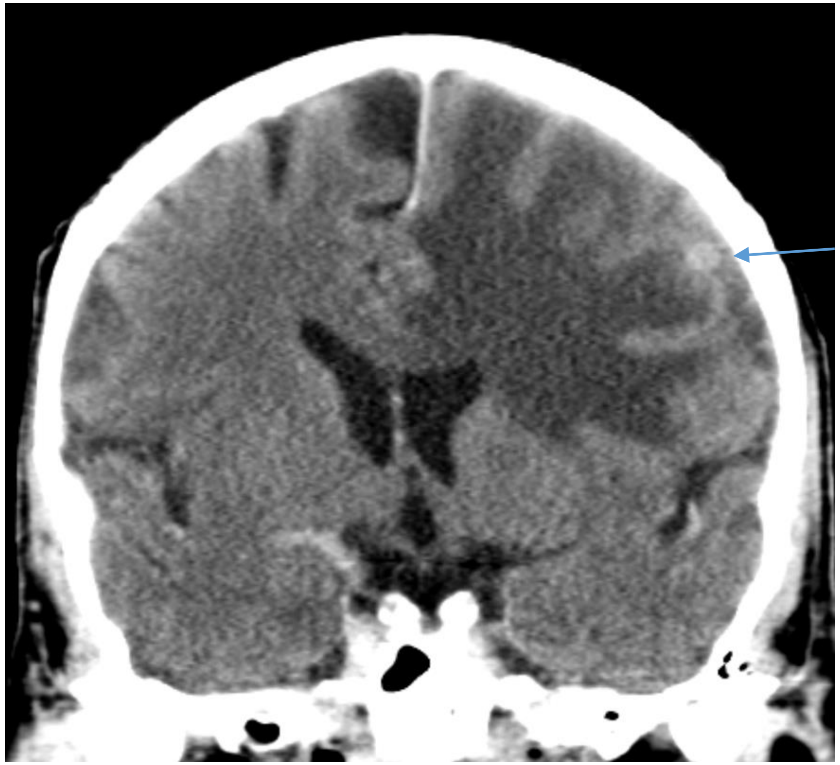

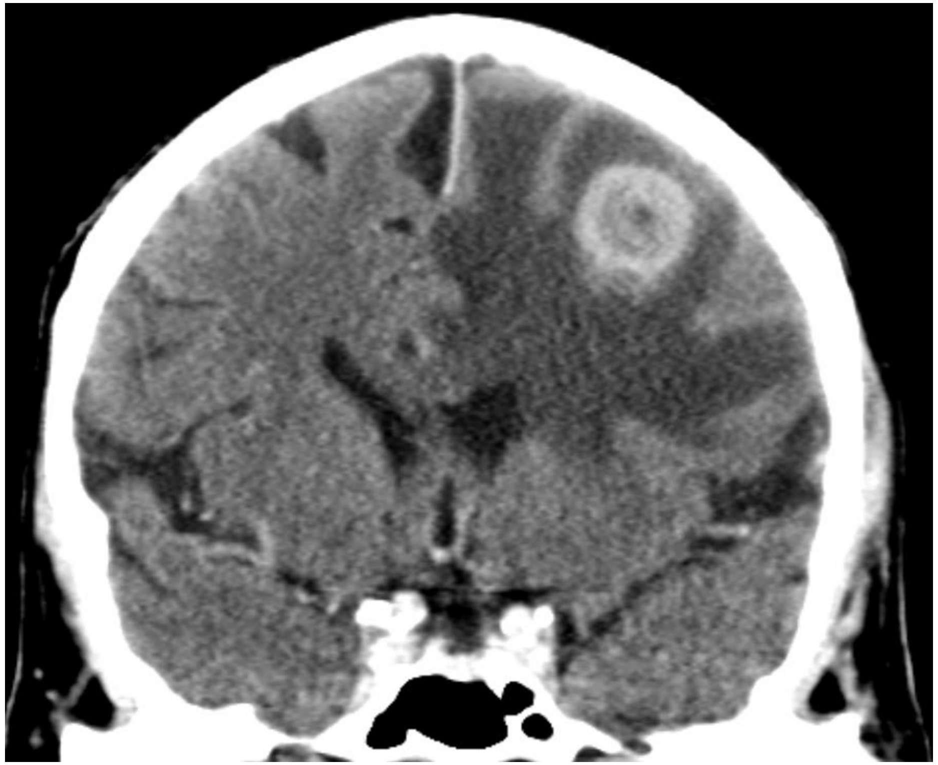

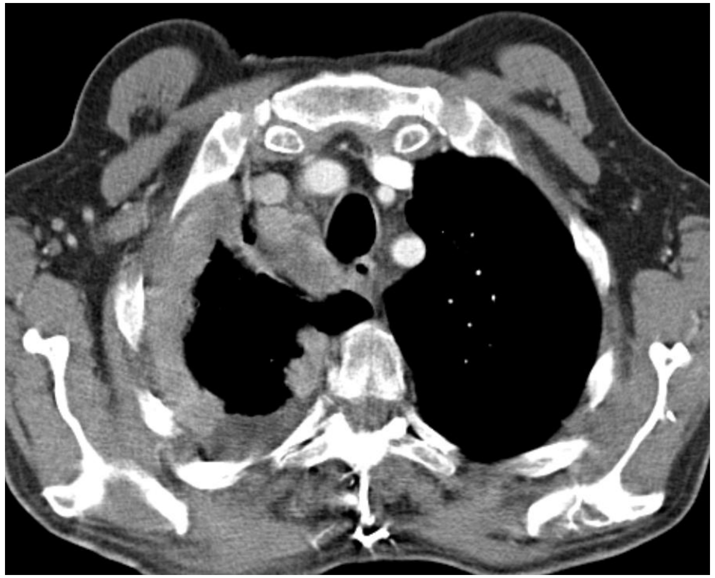

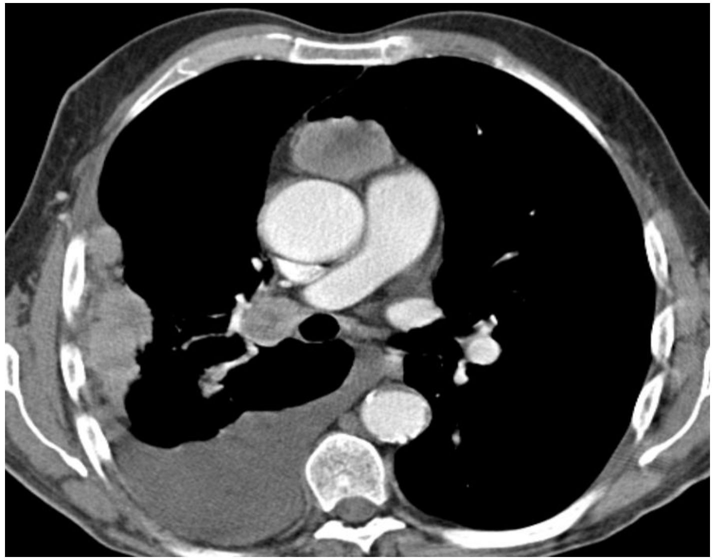

Patient 10: 6 brain metastases bilateral supratentorial, lung controlled, no other metastases

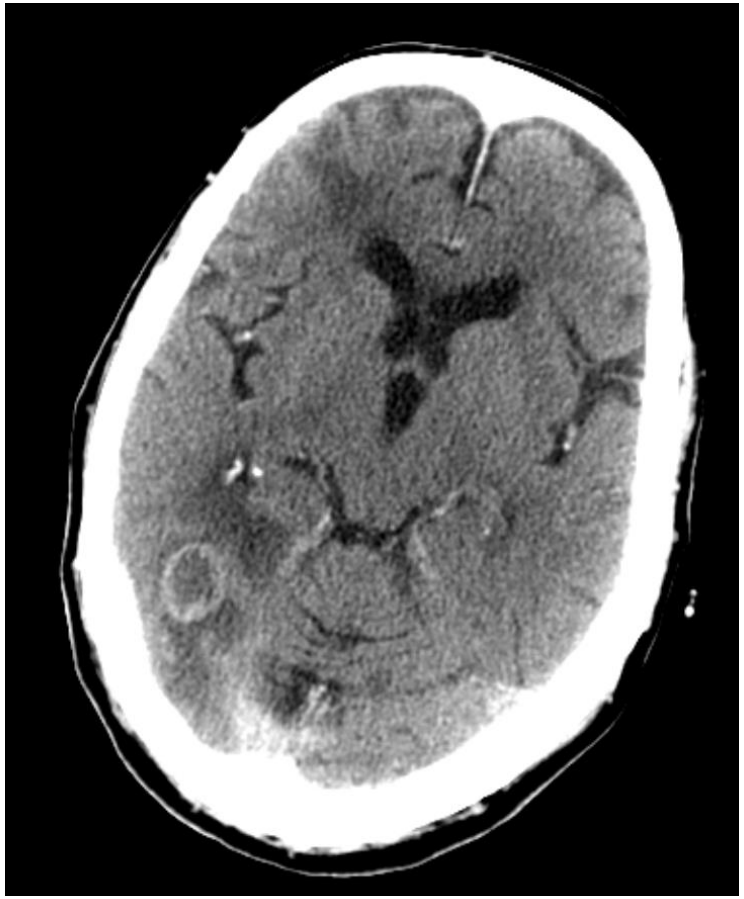

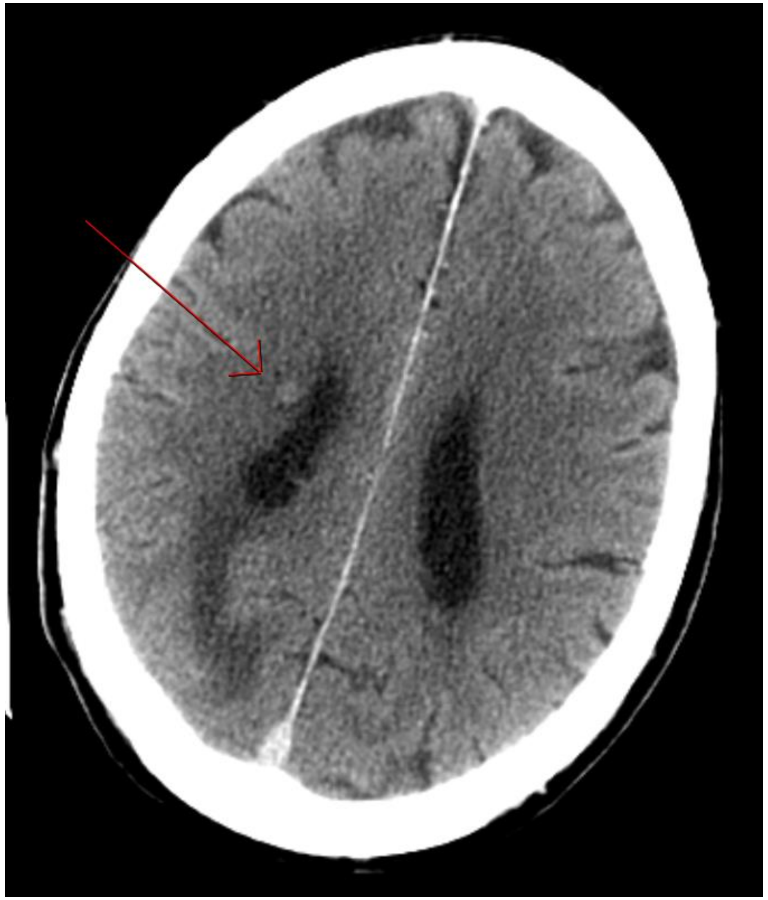

Patient 11: 5 brain metastases, lung controlled, adrenal gland metastases

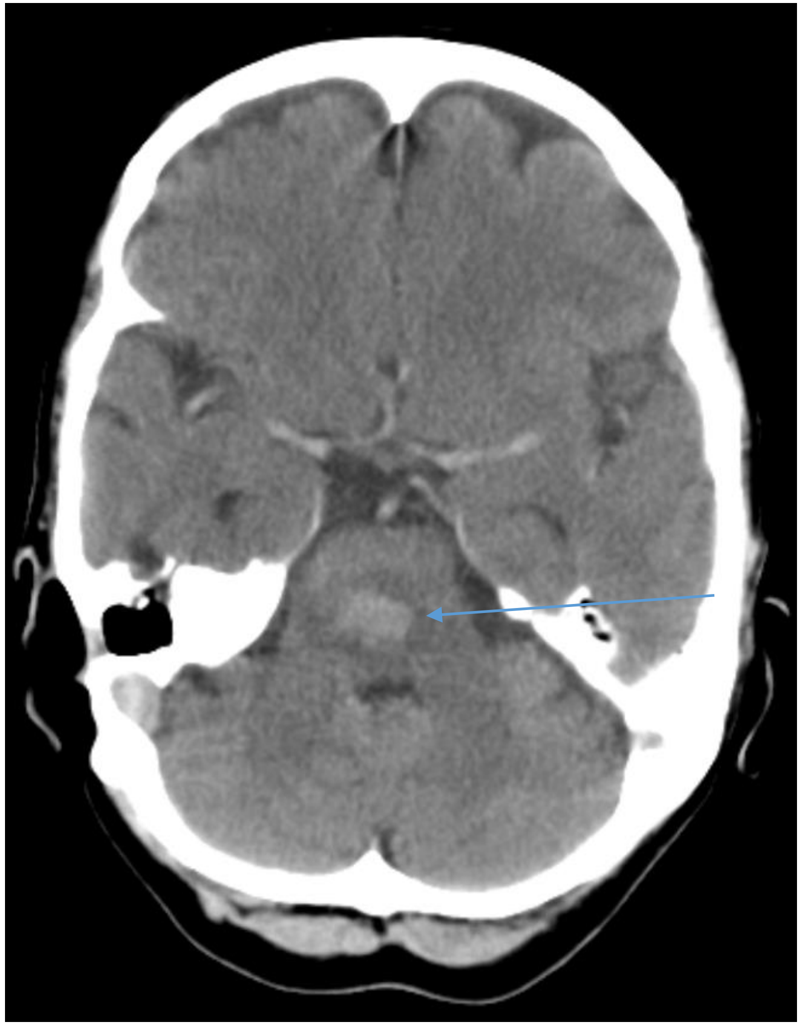

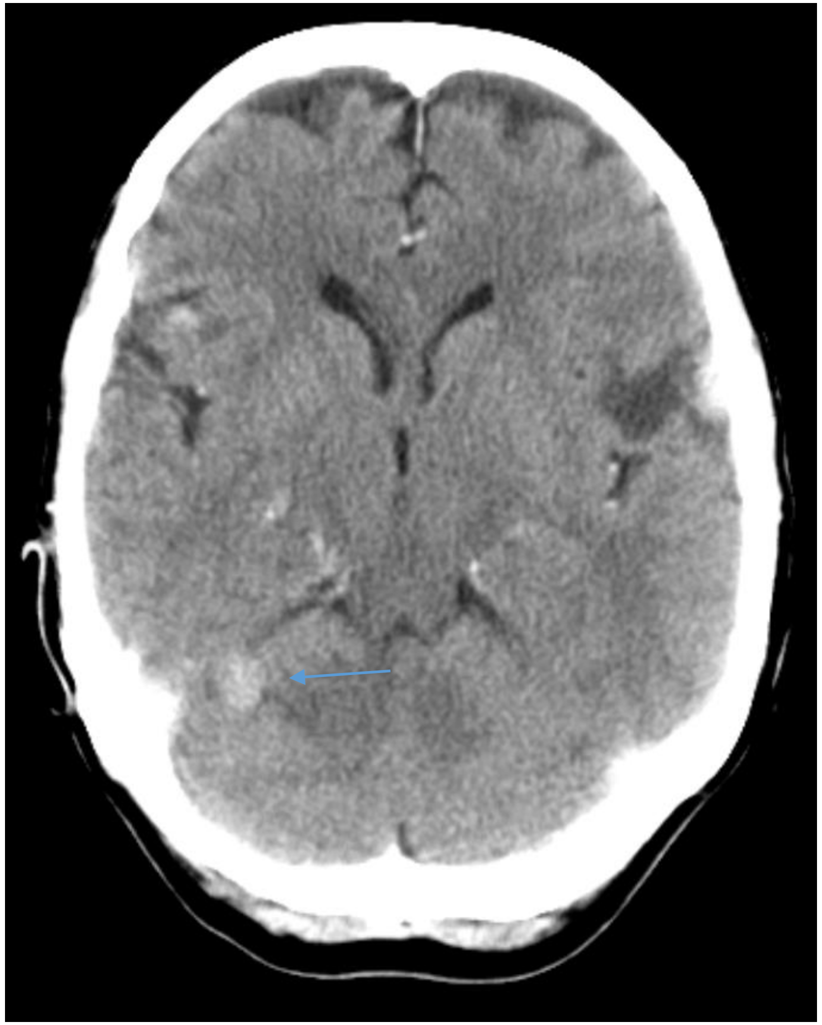

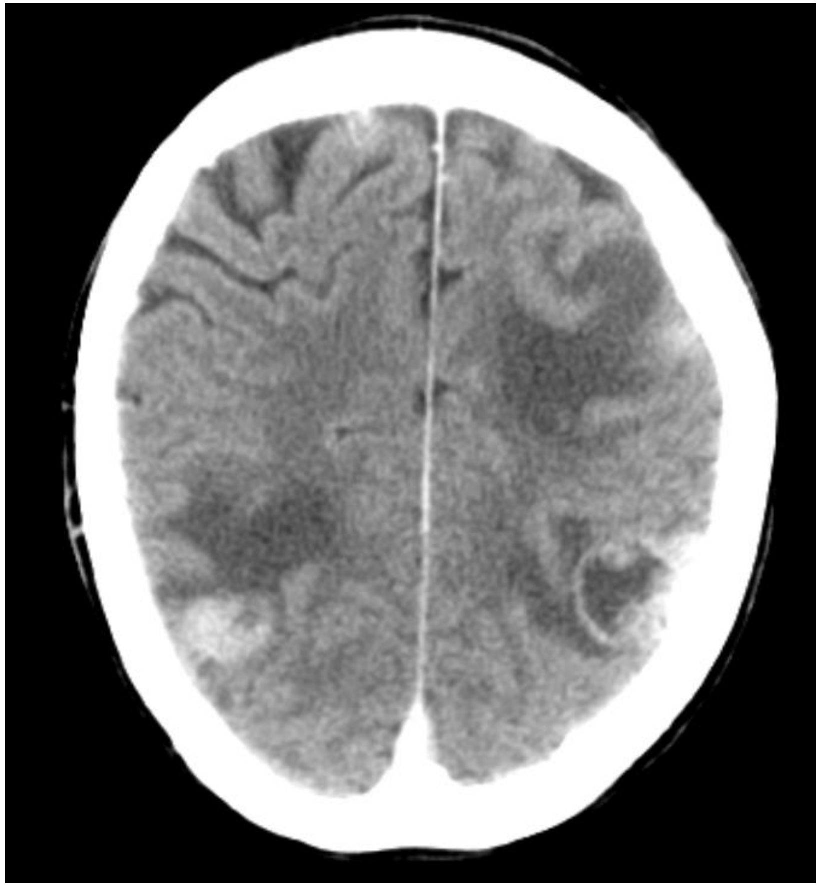

Patient 12: 4 brain metastases, lung controlled, bone metastases

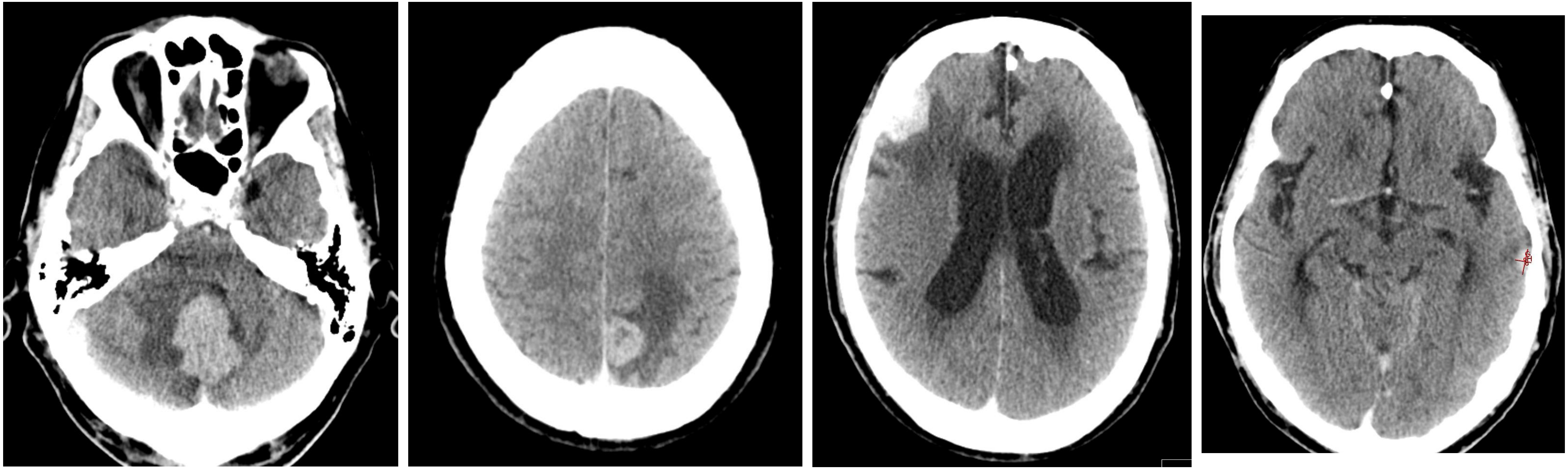

Patient 13: 7 brain metastases (2 infratentorial), lung controlled, several other distant metastases

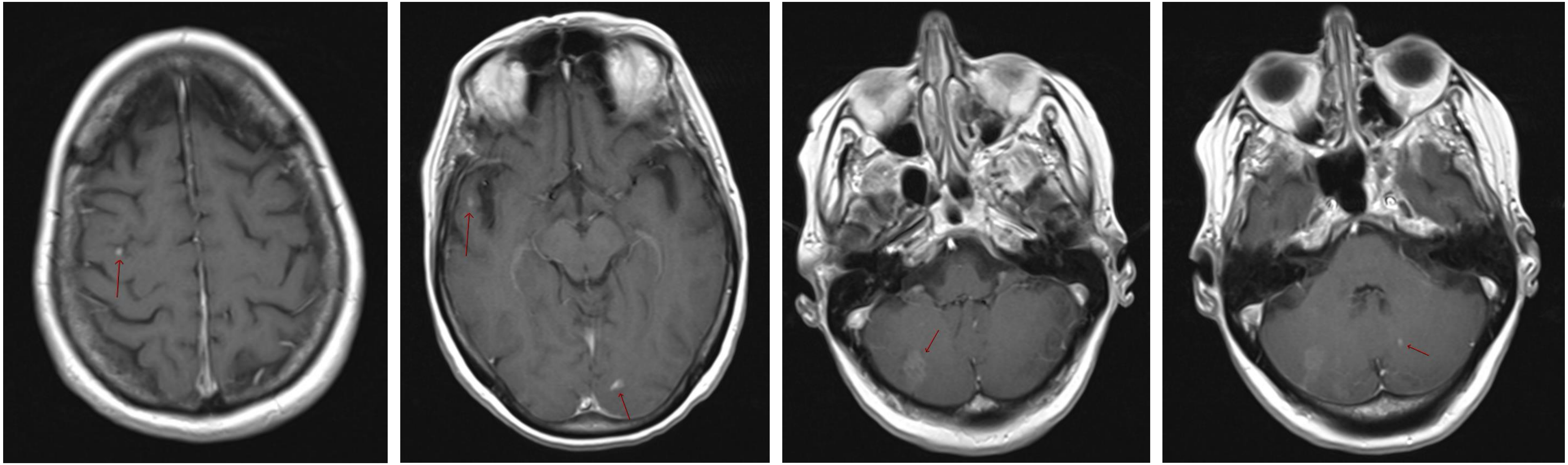

Patient 14: 3 brain metastases, lung uncontrolled local stage IIIB and pleural/lung metastases

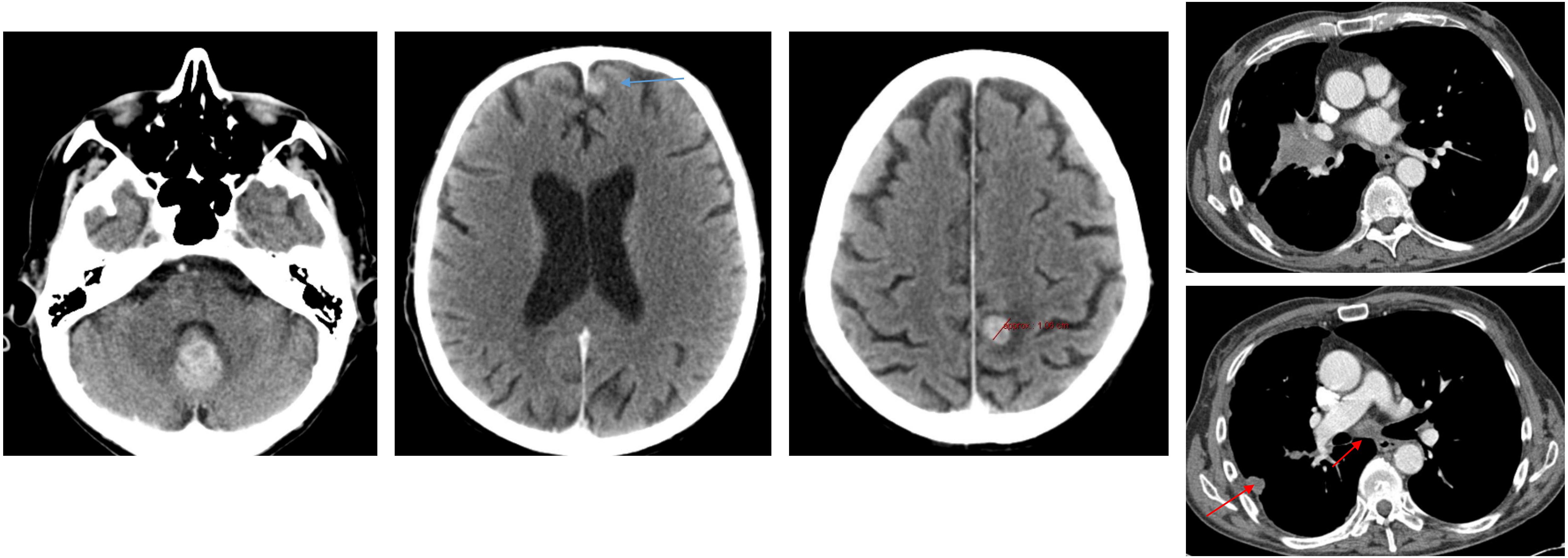

Patient 15: 18 brain metastases, lung controlled, several other distant metastases

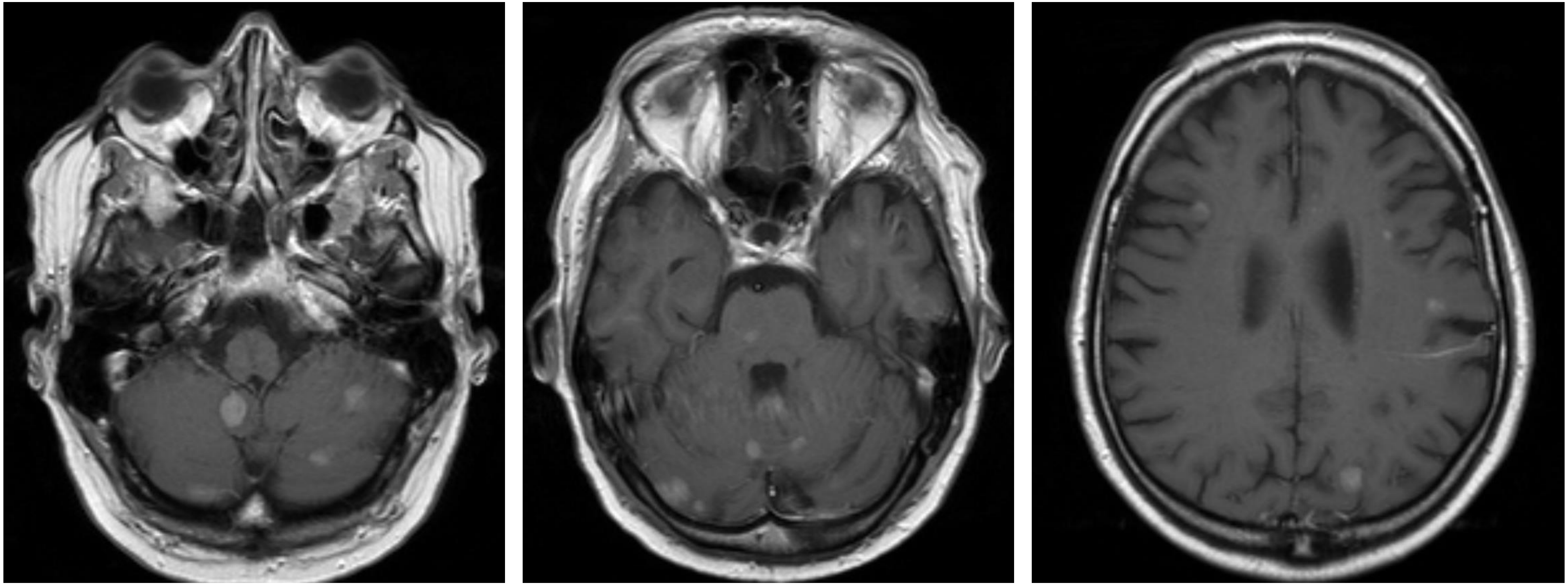

Patient 16: 4 brain metastases, lung controlled, lung metastases

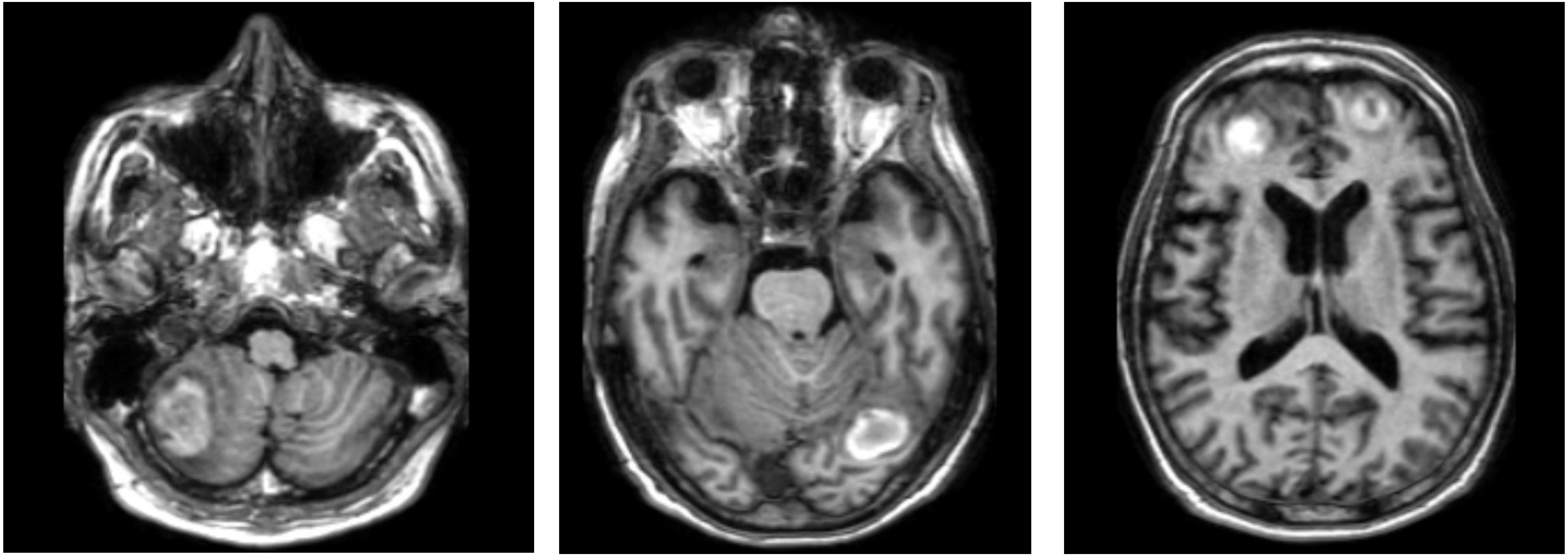

Patient 17: 7 brain metastases (all infratentorial), lung uncontrolled local stage IIIB, several other distant metastases

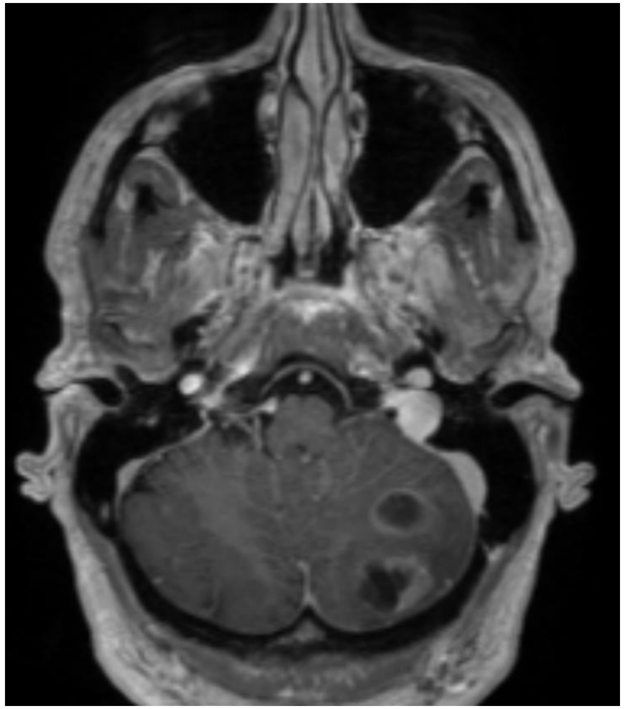
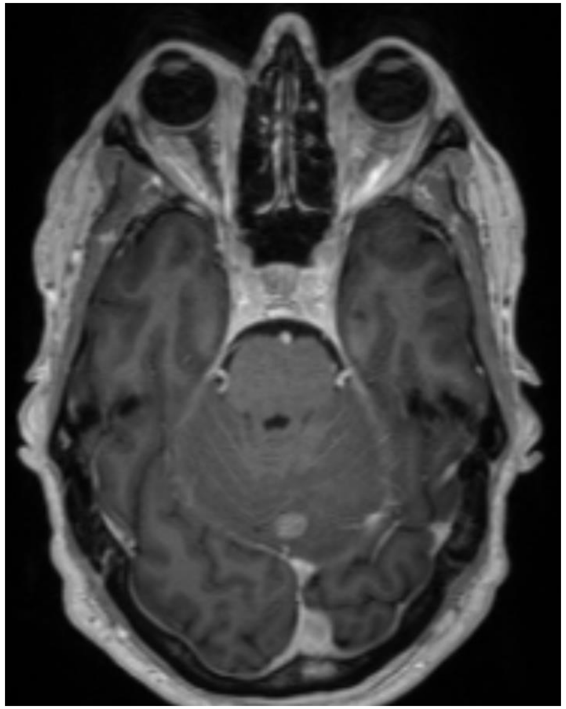
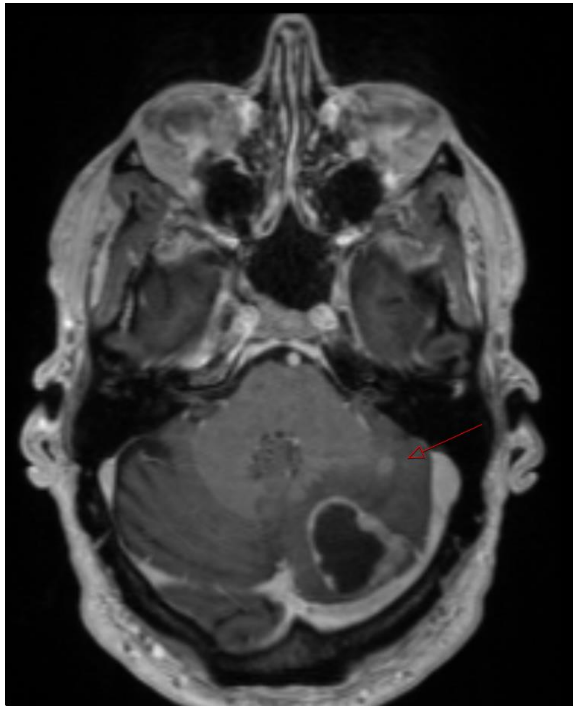
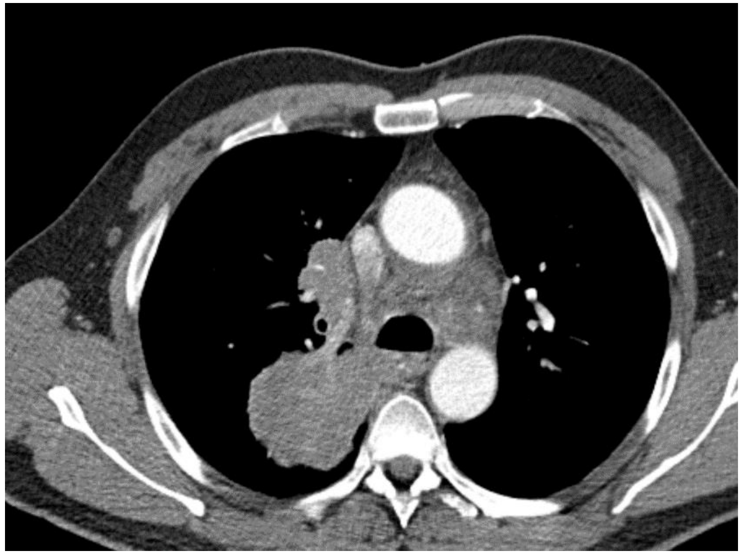
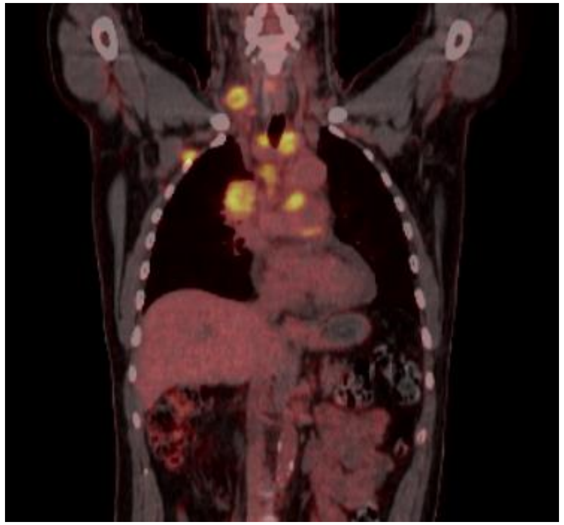

Supplement: Supplementary file 1 — Figure S1. Representative computed tomography and magnetic resonance imaging scans distributed to the participants. (PDF 3034 kb) [file 13014_2019_1237_MOESM1_ESM.pdf]
